# Allele-Specific Suppression of Mutant Huntingtin Using Antisense Oligonucleotides: Providing a Therapeutic Option for All Huntington Disease Patients

**DOI:** 10.1371/journal.pone.0107434

**Published:** 2014-09-10

**Authors:** Niels H. Skotte, Amber L. Southwell, Michael E. Østergaard, Jeffrey B. Carroll, Simon C. Warby, Crystal N. Doty, Eugenia Petoukhov, Kuljeet Vaid, Holly Kordasiewicz, Andrew T. Watt, Susan M. Freier, Gene Hung, Punit P. Seth, C. Frank Bennett, Eric E. Swayze, Michael R. Hayden

**Affiliations:** 1 Centre for Molecular Medicine and Therapeutics, Child and Family Research Institute, University of British Columbia, Vancouver, British Columbia, Canada; 2 ISIS Pharmaceuticals, Carlsbad, California, United States of America; 3 Behavioral Neuroscience Program, Department of Psychology, Western Washington University, Bellingham, Washington, United States of America; 4 Center for Advanced Research in Sleep Medicine, Department of Psychiatry, University of Montréal, Montréal, Quebec, Canada; University of Pittsburgh School of Medicine, United States of America

## Abstract

Huntington disease (HD) is an inherited, fatal neurodegenerative disorder caused by a CAG repeat expansion in the huntingtin gene. The mutant protein causes neuronal dysfunction and degeneration resulting in motor dysfunction, cognitive decline, and psychiatric disturbances. Currently, there is no disease altering treatment, and symptomatic therapy has limited benefit. The pathogenesis of HD is complicated and multiple pathways are compromised. Addressing the problem at its genetic root by suppressing mutant huntingtin expression is a promising therapeutic strategy for HD. We have developed and evaluated antisense oligonucleotides (ASOs) targeting single nucleotide polymorphisms that are significantly enriched on HD alleles (HD-SNPs). We describe our structure-activity relationship studies for ASO design and find that adjusting the SNP position within the gap, chemical modifications of the wings, and shortening the unmodified gap are critical for potent, specific, and well tolerated silencing of mutant huntingtin. Finally, we show that using two distinct ASO drugs targeting the two allelic variants of an HD-SNP could provide a therapeutic option for all persons with HD; allele-specifically for roughly half, and non-specifically for the remainder.

## Introduction

Huntington disease (HD) is an autosomal dominant, fatal neurodegenerative disorder with a prevalence of up to 17 cases per 100,000, which makes it one of the most common inherited neurodegenerative disorders [1], [2]. HD belongs to a family of polyglutamine diseases, and is caused by a mutation that expands a polyglutamine-encoding CAG repeat sequence in the huntingtin (*HTT*) gene [3]. The HTT protein is expressed ubiquitously and plays a central role in a plethora of interconnected cellular pathways [3]. The toxic effects mediated by mutant huntingtin (mHTT) are dependent on the number of CAG repeats in the gene, resulting in an inverse relationship between the age of symptom onset and the CAG repeat size [3]–[5]. The unaffected range is 6–35 CAG repeats, alleles with 36–39 CAGs confer increasing risk of developing HD, and alleles with 40 CAG repeats and above are fully penetrant, causing HD within normal lifespan [3], [6].

In 1983 the HD gene was mapped to the short arm of chromosome 4 and 10 years later the gene was isolated and cloned [7], [8]. Even though the mutation causing HD was discovered more than two decades ago and despite tremendous progress in our understanding of the mechanisms underlying HD, there is still no efficacious therapy available to prevent the disease. Current treatment relies solely on symptomatic relief, which is most often only satisfactory in the initial phase of the disease [9], [10]. Numerous drugs are being used to ameliorate the symptoms of HD including psychiatric agents, motor sedatives, and cognitive enhancers [10]. Only tetrabenazine has been approved by the FDA specifically to reduce the severity of chorea in HD [11]. Most of the potential therapeutic candidates which have been taken into clinical trials have had limited success [3]. These discouraging findings may be explained by the fact that most trials have only targeted one pathway in isolation and mHTT simultaneously disrupts multiple cellular pathways [3]. Therefore, preventing the expression of mHTT, which is the sole cause of disease, would be one of the most promising and comprehensive approaches for treating HD. Predictive testing and the identification of prodromal biomarkers in individuals positive for the HD mutation support the idea that preventative approaches are feasible [8], [12]. Furthermore, the likelihood of a successful outcome is good considering that treatment can be initiated early before detrimental changes occur. This belief is furthermore supported by multiple studies. For example, it has been shown that the expression level of mHTT correlates with the onset and progression of HD features in the YAC mouse model [13], suggesting that partial reduction of mHTT would be beneficial. Furthermore, it has been demonstrated, using a conditional HD mouse model, that HD phenotypes including neuropathology and motor symptoms can be reversed by turning the HD gene off [14].

Two different gene-silencing approaches are currently under development for HD. The first and most straightforward strategy is to suppress the expression of both the wild-type (wt) and mutant protein. However, a general concern for total HTT silencing has been raised regarding the potential side effects of reducing wtHTT, whose beneficial activity for neuronal function and maintenance is well established [3]. HTT is associated with several organelles and interacts with many molecular partners playing a critical role in numerous cellular processes including transcriptional regulation, protein homeostasis, oxidative stress, axonal transport, synaptic transmission, and apoptosis suppression [3]. It is currently not completely clear how much HTT is needed to maintain these functions in adulthood, but it has been shown that HTT has a crucial role during embryogenesis, since ablation of the Huntington Disease homolog (*Hdh*) gene in mice results in death at embryonic day 7–9 [15]–[17]. Reduction of wtHTT expression to about one third causes perinatal death and abnormal development of the CNS [18]. Moreover, one study shows that loss of half of wtHTT during development causes motor dysfunction, impaired behaviour and abnormal brain morphology and pathology [15]. Lastly, a conditional deletion in the forebrain of young adult mice leads to progressive neurodegeneration [19]. These findings demonstrate that wtHTT function is essential for brain development and neuronal survival and suggest that specific silencing of mHTT expression in adulthood may be a desirable choice. There are some studies conducted in HD mouse models that support the idea that reducing both wt and mHTT is well tolerated and leads to clinical benefit [20]–[23]. However, alterations in molecular pathways associated with loss of normal HTT function have also been observed [24], [25]. It is very difficult to predict how these findings may translate into human applications. Considering that HD patients would require life-long treatment and given the potential for side effects of long-term silencing of wtHTT, allele-specific strategies provide a valuable addition to the treatment options currently being considered.

Different approaches have been employed to achieve allele-specific silencing of mHTT by targeting disease-linked polymorphisms, including the CAG expansion [26]–[28], a CAG expansion-associated deletion [29], and single nucleotide polymorphisms (SNPs) enriched on HD alleles (HD-SNPs) [30]–[34]. Several CAG repeat-targeting silencing reagents are under pre-clinical development and have shown great promise when tested in cells from juvenile HD patients [28], [35]–[38], whom display a more severe form of the disease with onset before the age of 20 [39]. Juvenile HD accounts for less than 5–10% of HD patients [40] and it remains to be determined if this approach will be appropriate when the difference between the upper and lower CAG tracts is smaller, as with the majority of HD patients [41]. Some studies show that the selectivity of CAG targeted silencing reagents declines when the number of CAG repeats approaches the average size observed in the HD population [27], [38], [42], which suggests that this approach may not be beneficial to all HD patients [41]. Furthermore, CAG targeting strategies may be associated with the unwanted risk of reducing the expression of other CAG repeat containing transcripts, such as ataxin 3 [42], [43].

As an alternative therapeutic strategy, we and others have shown that targeting HD-SNPs using RNAi or antisense-oligonucleotides (ASOs) presents a promising approach towards achieving allele-specific treatment of HD patients [32]–[34], [44]. The heterozygosity of the SNPs in the HD population will determine the number of patients that will be amendable to this treatment strategy [41]. Since clinical trials are a considerable investment, selecting and evaluating the individual HD-SNPs becomes critical to achieve the maximal patient coverage with the lowest number of targeted SNPs. In addition to being in linkage disequilibrium with the CAG expansion, a targetable HD-SNP should ideally be found at low frequency on the wt allele to provide great specificity [44]. We have previously genotyped 234 Caucasian HD patients using a custom SNP genotyping assay and identified fifty HD-SNPs across the *HTT* gene that are significantly enriched on HD alleles compared to wt alleles [33], [44]. Out of these, forty are heterozygous in greater than 35% of the sequenced HD population, making them potential allele-specific silencing targets. ASOs against twenty-four of these HD-SNPs have previously been screened in primary human HD fibroblasts for mRNA knock down [33]. The top candidates were counter screened for protein knock down and the best candidate displayed approximately 70% knock down of mHTT in primary neurons from BACHD mice without affecting wtHTT protein levels in primary neurons from YAC18 mice [33]. The maximal coverage, which can be achieved by targeting one of these HD-SNP is roughly half of the HD population [33]. Population genetics studies show that 75%–85% of the HD population could be treated with panel of three to five ASOs targeting these HD-SNPs [32], [44]. Therefore, in addition to selecting a primary HD-SNP target, it becomes important to include supplementary HD-SNPs, which are not in linkage disequilibrium, to increase patient coverage. The majority (∼90%) of the identified HTT SNPs are intronic and can only be targeted by ASOs that, unlike RNAi, do not require the endogenous microRNA processing machinery for activity [45]. ASOs promote RNase H-induced cleavage of pre-mRNA and mature mRNA preventing the generation of protein [46]. ASOs are freely taken up by neurons and can be delivered to the CNS via intrathecal injections or infusions, allowing for a rapid and controlled dosing strategy [23], [47], [48], making ASOs attractive candidates for therapeutic intervention.

ASO-mediated HTT knock down was demonstrated more than a decade ago using both phosphodiester and phosphorothiorated (PS) ASOs [49], [50]. Since that time, the development of ASO technology has steadily progressed in both research and clinical settings. Research has focused on ASO designs that increase resistance to degradation, improve affinity and enhance specificity, thereby increasing potency and reducing undesirable off-target effects. Here, we have established a functional pipeline that allows for rapid screening and selection of potent, selective, and well tolerated ASOs in primary neurons. For our screen, we have used neurons from the humanized Hu97/18 mouse, which has human wt and mHTT transgenes, along with the corresponding SNPs associated with each human allele, and no endogenous murine Hdh [51]. Here, we evaluate both previously reported and novel ASOs in a system pertinent to the brain using a novel triage system based on protein knock down, selectivity, and toxicity to select well tolerated ASOs providing the greatest mHTT knock down while maintaining normal expression of wtHTT. This approach has resulted in identification of several promising leads and progress towards a therapeutic option for all HD patients and the screening strategy could be adapted for identification of therapeutic ASOs for other indications where allele-specific knockdown would be beneficial.

## Results

### ASO screening pipeline

**Figure 1:**
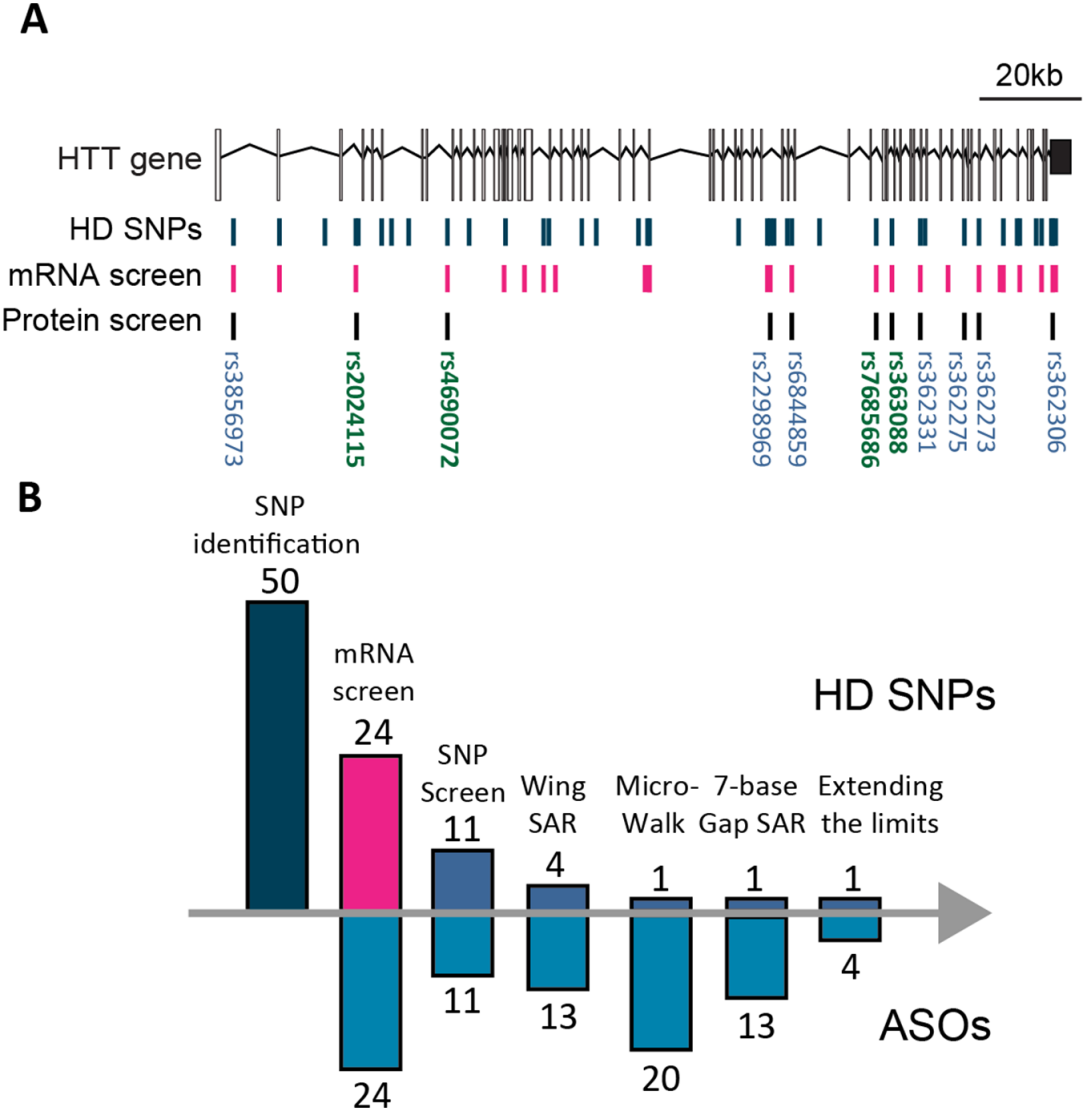
ASO screening pipeline. (A) HD-SNPs in the *HTT* gene: blue = HD-SNPs, pink = previous human fibroblasts screen, grey = Hu97/18 screen; green Rs numbers = SNPs identified as the most RNase-H-active sites (B) ASO development pipeline: The number of targeted SNPs and ASOs tested are shown above and below the column bars, respectively. 50 SNPs are enriched on HD alleles and ASOs targeting 24 of these were previously screened for mHTT mRNA silencing. ASOs targeting 10 SNPs were screened in primary Hu97/18 neurons for HTT protein suppression and tolerability. Then, ASOs with modifications to the wings targeting 4 of these SNPs were screened. Microwalk SAR and 7-base gap SAR was done for oligos targeting SNP Rs7685686. Lastly, higher ASO concentrations and longer treatment durations were tested.

Out of the fifty HD-SNPs previously identified [33], [44], ten SNPs were selected as a starting point for efficacy studies in primary Hu97/18 neurons based on therapeutic relevance and availability of screening tools (Figure 1A). These SNPs are each heterozygous and targetable (present on the CAG expanded chromosome) in greater than 35% of the sequenced HD population as well as in available HD patient-derived fibroblast cell lines and the Hu97/18 mouse model of HD. Single ASOs were tested at ten different SNPs and the four most active ASOs were moved forward (Figure 1B). We employed three different structure-activity relationship (SAR) studies to find the best possible ASO candidates. The first approach was to change the number and position of modifications in the wings of the ASO. Next, we conducted a microwalk of the sequence around the target SNP site and lastly, we have evaluated the effect of shortening the ASO gap from 9 to 7 nucleotides. ASOs were screened for potency and specificity. Additionally, to exclude toxic ASOs from the pipeline, we used cleavage of spectrin, a cytoskeletal protein that lines the intracellular surface of the plasma membrane and is cleaved by caspases during apoptosis [52], as a measure of neuronal tolerability.

### Identification of the best targetable SNPs

The ultimate goal is to develop a panel of allele-specific ASOs that, in combination, will provide a therapeutic option to the majority of the HD patients. However, the purpose of this screen was to identify the most efficacious SNP sites and to develop the best possible ASO candidate. The selected HD-SNPs in the current study do not provide significant combinatorial advantage as they are all in high linkage disequilibrium with one another. To evaluate the activity at several SNP sites we used phosphorothioate substituted 19-mers containing five 2′-O-methoxy-ethyl (MOE; represented by “e”) ribose sugars in each wing and a string of nine DNA residues in the gap (5e-9-5e gapmers) [53]. Primary Hu97/18 cortical neurons were treated with bath-applied ASO on the second day in culture at 0, 16, 62.5, 250, and 1000 nM. After 6 days of treatment, the neurons were collected and the two allelic proteins were separated by electrophoresis based on differences in CAG size and assessed by Western blotting. The HTT protein was quantified and normalized to the calnexin loading control. Subsequently, membranes were re-blotted for spectrin, and the amount of cleaved spectrin (120 kDa fragment) was evaluated as an apoptosis readout with a toxicity threshold of 3-fold induction. As a positive control for spectrin cleavage, we used camptothecin, which is a topoisomerase inhibitor causing DNA damage, to induce apoptosis (Figure S1) [52], [54]. Dose-response curves were generated for HTT knock down and the specificity was determined by calculating the ratio of the IC_50_ values for wtHTT and mHTT. If there was less than 50% knock down at the highest ASO concentration tested and no possibility of calculating an IC_50_ value, then the highest ASO concentration evaluated was used to calculate the ratio, which is expressed as >x fold.

**Figure 2:**
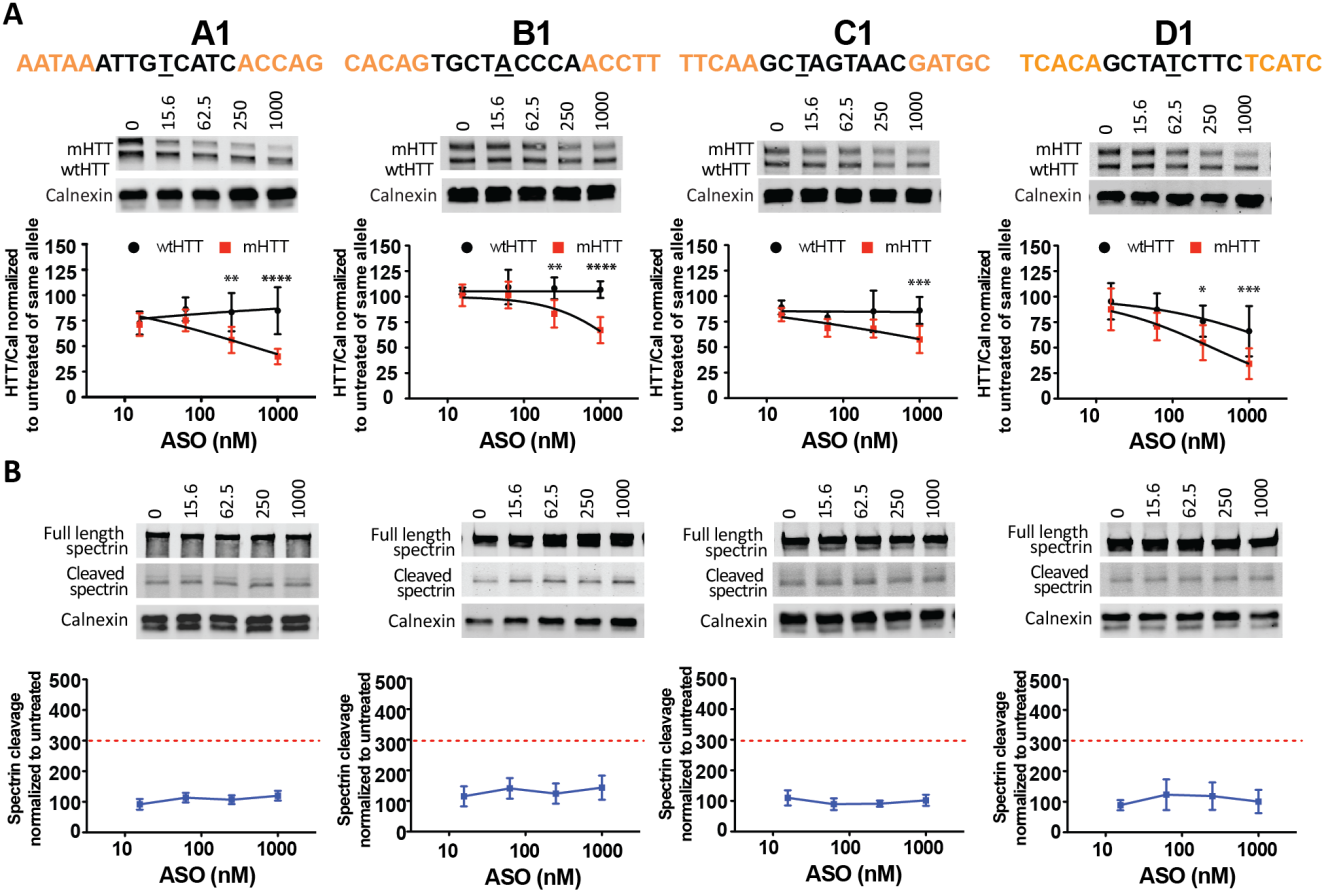
Selection of the best SNP targets. Primary Hu97/18 neurons were treated with 5e-9-5e ASOs targeted to 10 HD-SNPs at 6–1000 nM for 6 days. (A) HTT Western blots and quantitation for the 4 SNPs with the greatest activity. HTT levels are normalized to the internal loading control calnexin and then to the untreated sample for each allele. (B) Western blots showing full length and cleaved spectrin for the 4 ASOs. Spectrin fragment is normalized to calnexin and then to the untreated sample. Membranes were probed for HTT and reprobed for spectrin. Representative images are shown. n = 4–8 per data point. Data are presented as mean ± SD. Two way ANOVA with Bonferroni post hoc test have been performed and p values are illustrated with *, **, ***, **** for p = 0.05, 0.01, 0.001, and 0.0001. The PS backbone is black and MOE modifications are illustrated by orange. The SNP is underlined. The red dashed line represents the toxicity threshold.

ASOs A1, B1, C1, and D1 targeting rs7685686, rs4690072, rs2024115, rs363088, respectively, showed acceptable potency (34–79% mHTT remaining at 1000 nM), specificity up to 3.1 fold, and no overt toxicity (Figure 2 and Table 1 *)*. These 4 candidates were moved forward in the pipeline. The remaining ASOs showed limited HTT silencing and specificity that did not reach statistical significance with two-way ANOVA and Bonferroni post hoc test (Figure S2). None of the 10 ASOs tested displayed spectrin cleavage above threshold (Figure S3). However, treatment with ASOs E1 and G1 for 6 days caused marked morphological changes of the neurons with increasing severity with doses starting at 250 and 500 nM, respectively (Figure S4). These findings suggest that some adverse structural changes may be occurring, and these ASOs were therefore excluded. The fact that the ASOs, having the same chemistry and design, are not equally active at all SNP sites, suggests that the location of the target SNP within the pre-mRNA sequence may play a critical role for its accessibility. It could be speculated that secondary structures in the pre-mRNA may prevent efficient ASO binding and RNase H recruitment to certain sequences.

**Table 1:** Summary of ASO protein screen in Hu97/18 primary neurons.

ASO	Notation	Rs #	Max effect		Day	IC50		Selectivity	Toxicity	Screen
			%wtHTT	%mHTT		wtHTT	mHTT			
**A1**	AeAeTeAeAeATTGTCATCAeCeCeAeGe	rs7685686	85	40	D6	>1000	421	>2.4	Pass	**Pass**
**B1**	CeAeCeAeGeTGCTACCCAAeCeCeTeTe	rs4690072	109	79	D6	>1000	>1000	ND	Pass	**Pass**
**C1**	TeTeCeAeAeGCTAGTAACGeAeTeGeCe	rs2024115	86	57	D6	>1000	>1000	ND	Pass	**Pass**
**D1**	TeCeAeCeAeGCTATCTTCTeCeAeTeCe	rs363088	66	34	D6	>1000	330	>3.1	Pass	**Pass**
**E1**	AeAeGeGeGeATGCTGACTTeGeGeGeCe	rs2298969	96	72	D6	>1000	>1000	ND	Pass	-
**F1**	CeCeTeTeCeCTCACTGAGGeAeTeGeAe	rs6844859	40	25	D6	538	234	2.3	Pass	-
**G1**	GeCeAeCeAeCAGTAGATGeAeGeGeGeAe	rs362331	80	70	D6	>1000	>1000	ND	Pass	-
**H1**	AeAeGeAeAeGCCTGATAAAeAeTeCeTe	rs362275	82	65	D6	>1000	>1000	ND	Pass	-
**I1**	GeAeGeCeAeGCTGCAACCTeGeGeCeAe	rs362306	52	43	D6	>1000	635	>1.6	Pass	-
**J1**	TeTeGeAeTeCTGTAGCAGCeAeGeCeTe	rs362273	94	84	D6	>1000	>1000	ND	Pass	-
**A2**	AeTkAeAkATTGTCATCAkCeCkAe	rs7685686	22	7	D6	74	9	8.2	-	-
**A3**	TeAkAkATTGTCATCAkCkCe	rs7685686	28	9	D6	356	40	8.9	Pass	**Pass**
**B2**	AeCkAeGkTGCTACCCAAkCeCkTe	rs4690072	53	35	D6	>1000	280	>3.6	-	-
**B3**	CeAkGkTGCTACCCAAkCkCe	rs4690072	57	34	D6	>1000	272	>3.6	-	-
**C2**	CeTkTeCkAAGCTAGTAAkCeGkAe	rs2024115	93	53	D6	>1000	>1000	ND	Pass	-
**C3**	TeTkCkAAGCTAGTAAkCkGe	rs2024115	81	44	D6	>1000	538	>1.8	Pass	-
**D2**	CeAkCeAkGCTATCTTCTkCeAkTe	rs363088	63	32	D6	>1000	262	>3.8	-	-
**D3**	AeCkAkGCTATCTTCTkCkAe	rs363088	52	33	D6	>1000	185	>5.4	-	-
**A4**	AeAkTeAkAeATTGTCATCAeCeCeAeGe	rs7685686	20	6	D6	57	13	4.4	Pass	-
**A5**	AkAeTkAeAkATTGTCATCAkCeCkAeGk	rs7685686	27	9	D6	139	14	9.9	-	-
**A6**	AkATkAAkATTGTCATCAkCCkAGk	rs7685686	20	7	D6	57	7	8.1	Pass	-
**A7**	AeTkAkAkATTGTCATCAkCkCkAe	rs7685686	14	5	D6	46	11	4.2	-	-
**A8**	AkTeAkAkATTGTCATCAkCkCeAk	rs7685686	14	4	D6	64	14	4.6	-	-
**A11**	AeAkTkTkGTCATCACCAkGe	rs7685686	27	7	D6	149	11	13.5	-	-
**A20**	TkTeGTCATCACCAkGkAkAe	rs7685686	24	8	D6	207	23	9.0	-	-
**A21**	AeTkTGTCATCACCkAkGkAe	rs7685686	52	11	D6	>1000	48	>21	-	-
**A22**	AeAkTTGTCATCACkCkAkGe	rs7685686	61	18	D6	>1000	78	>12.9	Pass	**Pass**
**A29**	AeTeAeAeAeTTGTCATCeAeCeCeAe	rs7685686	82	52	D6	>1000	>1000	ND	Pass	-
**A30**	AkTkAkAkAkTTGTCATCkAkCkCkAk	rs7685686	70	20	D6	>1000	38	>26.3	-	-
**A31**	AeTeAeAeAkTkTGTCATCAkCkCeAe	rs7685686	78	18	D6	>1000	94	>10.6	-	-
**A32**	AeTkAeAkAeTkTGTCATCAkCeCkAe	rs7685686	63	24	D6	>1000	77	>13.0	-	-
**A33**	AeTeAeAkAkTTGTCATCkAkCeCeAe	rs7685686	58	25	D6	>1000	74	>13.5	Pass	-
**A34**	AeTeAeAeAkTkTGTCATCAkCeCkAe	rs7685686	50	13	D6	>1000	18	>55.5	-	-
**A35**	TeAeAeAkTkTGTCATCAkCkCeAeGe	rs7685686	76	18	D6	>1000	42	>23.8	Pass	-
**A36**	TeAeAeAeTkTGTCATCAkCeCeAe	rs7685686	109	32	D6	>1000	254	>3.9	Pass	-
**A37**	TeAeAeAkTkTGTCATCAkCkCeAe	rs7685686	70	17	D6	>1000	67	>14.9	-	-
**A38**	AeTeAeAeAkTkTGTCATCAkCkCe	rs7685686	86	16	D6	>1000	31.9	>31.3	Pass	**Pass**
**A39**	TeAeAeAkTkTGTCATCAkCkCe	rs7685686	82	14	D6	>1000	38.4	>26.0	Pass	**Pass**
**A40**	TeAkAkATkTGTCATCAkCkCe	rs7685686	87	25	D6	>1000	121.9	>8.2	Pass	**Pass**
**A41**	TeAkAkAkTkTGTCATCAkCkCe	rs7685686	72	19	D6	>1000	44.6	>22.4	Pass	**Pass**
**A38**	AeTeAeAeAkTkTGTCATCAkCkCe	rs7685686	82	10	D10	>1000	16.1	>62.1	Pass	**Pass**
			71	7	D15	>1000	6.8	>147.1	Pass	
			72	17	D6High	>10000	77	>130.0	Pass	
**A39**	TeAeAeAkTkTGTCATCAkCkCe	rs7685686	63	9	D10	>1000	17.0	>58.8	Pass	**Pass**
			60	6	D15	>1000	9.8	>102	Pass	
			81	16	D6High	>10000	68	>147.1	Pass	
**A40**	TeAkAkATkTGTCATCAkCkCe	rs7685686	89	20	D10	>1000	52.7	>19.0	Pass	-
			63	9	D15	>1000	17.6	>66.8	-	
			70	23	D6High	>10000	166.	>60.0	-	
**A41**	TeAkAkAkTkTGTCATCAkCkCe	rs7685686	79	12	D10	>1000	8.1	>123.5	-	-
			38	6	D15	632	5.6	113	-	
			53	15	D6High	8919	67	133	-	
**X1**	AeTeAeAeAkTkTGCCATCAkCkCe	rs7685686	97	35	D6	ND	150	ND	Pass	**Pass**
**X2**	TeAeAeAkTkTGCCATCAkCkCe	rs7685686	103	34	D6	ND	134	ND	Pass	**Pass**

MOE and cEt modifications are annotated by e and k, respectively. The SNP is underlined. Maximal effect at highest dose. IC50, half maximal inhibitory concentration (nM).

### ASO wing SAR screen

**Figure 3:**
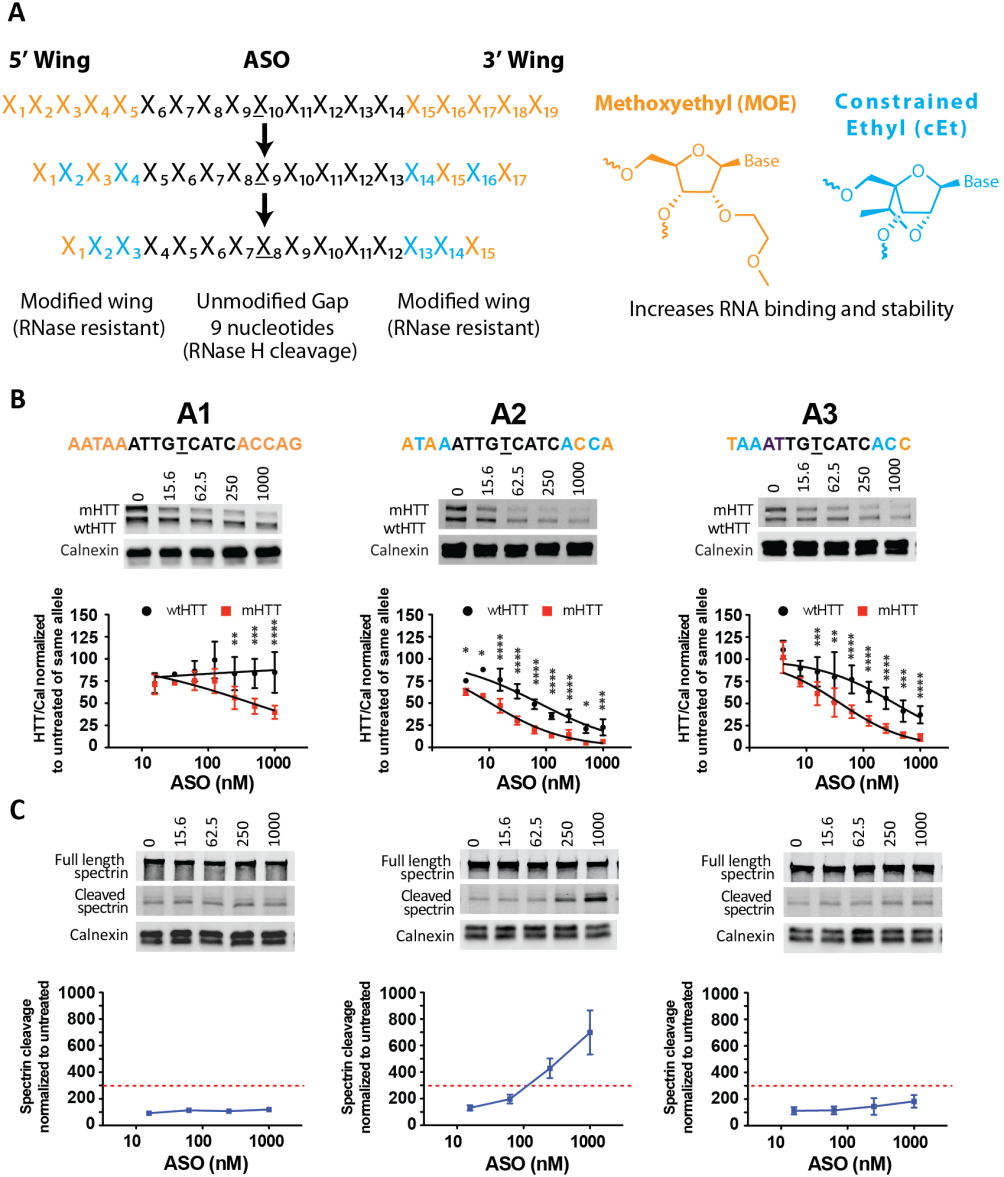
ASO screen at 4 SNPs using two different cEt motifs. (A) ASOs with two different cEt-modified wing motifs (ekek-9-keke and ekk-9-kke) were compared to the parent MOE oligos (5e-9-5e). Primary Hu97/18 neurons were treated with ASO at 1–1000 nM for 6 days. (B) HTT Western blot and quantitations. HTT levels are normalized to the internal loading control calnexin and then to the untreated sample for each allele. (C) Western blots showing full length and cleaved spectrin. Spectrin fragment is normalized to calnexin and then to the untreated sample. Membranes were probed for HTT and reprobed for spectrin. Representative images are shown. n = 6–8 per data point. Data are presented as mean ± SD. Two way ANOVA with Bonferroni post hoc test have been performed and p values are illustrated with *, **, ***, **** for p = 0.05, 0.01, 0.001, and 0.0001. The PS backbone is black, MOE and cEt modifications are illustrated by orange and blue, respectively. The SNP is underlined. The red dashed line represents the toxicity threshold.

To further evaluate ASOs targeting these four SNPs and identify the ASOs with the most activity, we introduced S-constrained-ethyl (cEt; represented by “k”) modifications to the wings of the 4 parent ASOs A1, B1, C1, and D1. Prior studies using these high affinity modifications in the wings found ASOs displaying improved potency without producing toxicity [55], [56]. Similarly, we have found that the introduction of cEt modifications increases the potency of the ASOs significantly when used in primary neurons [33]. We have tested ASOs of three different lengths (19, 17, and 15 nucleotides) and with the incorporation of two different wing motifs, ekek-9-keke and ekk-9-kke (Figure 3A). We believed that it would be more achievable to identify ASOs with sufficient potency and then subsequently improve specificity than to try to enhance the potency of a highly specific ASO. Therefore, for the ASO to move forward and pass this secondary screen, we focused on identifying tolerable oligos (no visual morphological changes and less than 3-fold induction of spectrin cleavage) with good potency (IC_50,mHTT_<200 nM) and moderate specificity (>5 fold). The most promising ASO series: A1, A2, and A3, is shown as an example in figure 3. We found 40 and 10 fold increase in potency for A2 and A3, respectively, compared to A1 (Figure 3B). Since the specificity for A1 (>2.4) was calculated by using the highest dose tested, it cannot be directly compared to A2 and A3 that showed allele-specific silencing of mHTT by 8.2 and 8.9 fold respectively (Table 1). A2 exhibited above threshold spectrin cleavage and was excluded, whereas the shortest molecule of the series, A3, did not show overt toxicity (Figure 3C). The remaining ASOs did not pass our selection criteria (Table 1, Figure S5 and S6), and A3 was the only ASO of the eight evaluated candidates to move forward.

In parallel, we wanted to investigate whether adding additional cEt modification to the wings of the ASO would lead to improvement in potency and specificity. To evaluate this, while maintaining the 9-base gap, three 19mer oligos, A4, A5, and A6, based on A1 were evaluated. First, we added two cEt modifications to the 5′ wing (A4; ekeke-9-5e) while keeping the 3′ wing only modified with MOE chemistry. Next, we mixed the modifications in both wings (A5; kekek-9-kekek), and finally replaced the MOE modifications with unmodified PS deoxynucleotides (represented by “d”) and alternating cEt modifications (A6; kdkdk-9-kdkdk). All ASOs displayed excellent potency (IC_50,mHTT_<14 nM) (Table 1). A4 showed reduced specificity compared to A3, whereas A5 and A6 showed comparable specificity of 9.9 and 8.1 fold, respectively. For A5 the small increase in potency and specificity unfortunately came at the cost of spectrin cleavage above threshold (Figure S7). Lastly, we evaluated two ASOs, A7 and A8, based on A2. While A2 did not meet the tolerability criteria from the previous screen, it demonstrated extremely high potency, which is an attractive property for a potential therapeutic. Greater potency translates to lower therapeutic doses, thus reducing cost and potentially reducing side effects. ASOs A7 and A8 were generated in an effort to determine if changes to the wing motif could mitigate the toxic effects of A2 while maintaining the superior potency. First, one cEt modification was added to each wing (A7; ekkk-9-kkke) and from this design the MOE and the cEt modifications were switched in each wing (A8; kekk-9-kkek). The two ASOs, A7 and A8, had a similar profile to the parent molecule, A2, displaying excellent potency (IC_50,mHTT_<14 nM), but with a small reduction in specificity (4.2 and 4.6 fold). Both ASOs induced spectrin cleavage above threshold and were therefore excluded. The strategy of changing and rearranging modifications of the wings with MOE and cEt nucleotides did not provide an ASO with a better profile than A3. Overall, our primary screen identified one tolerable candidate, A3, with good potency and moderate specificity. While it did not demonstrate the best specificity, we thought it would be easier to improve specificity with chemical modification than to improve potency. A3 was therefore used as the parent molecule for the subsequent SAR studies.

### SNP Microwalk SAR

**Figure 4:**
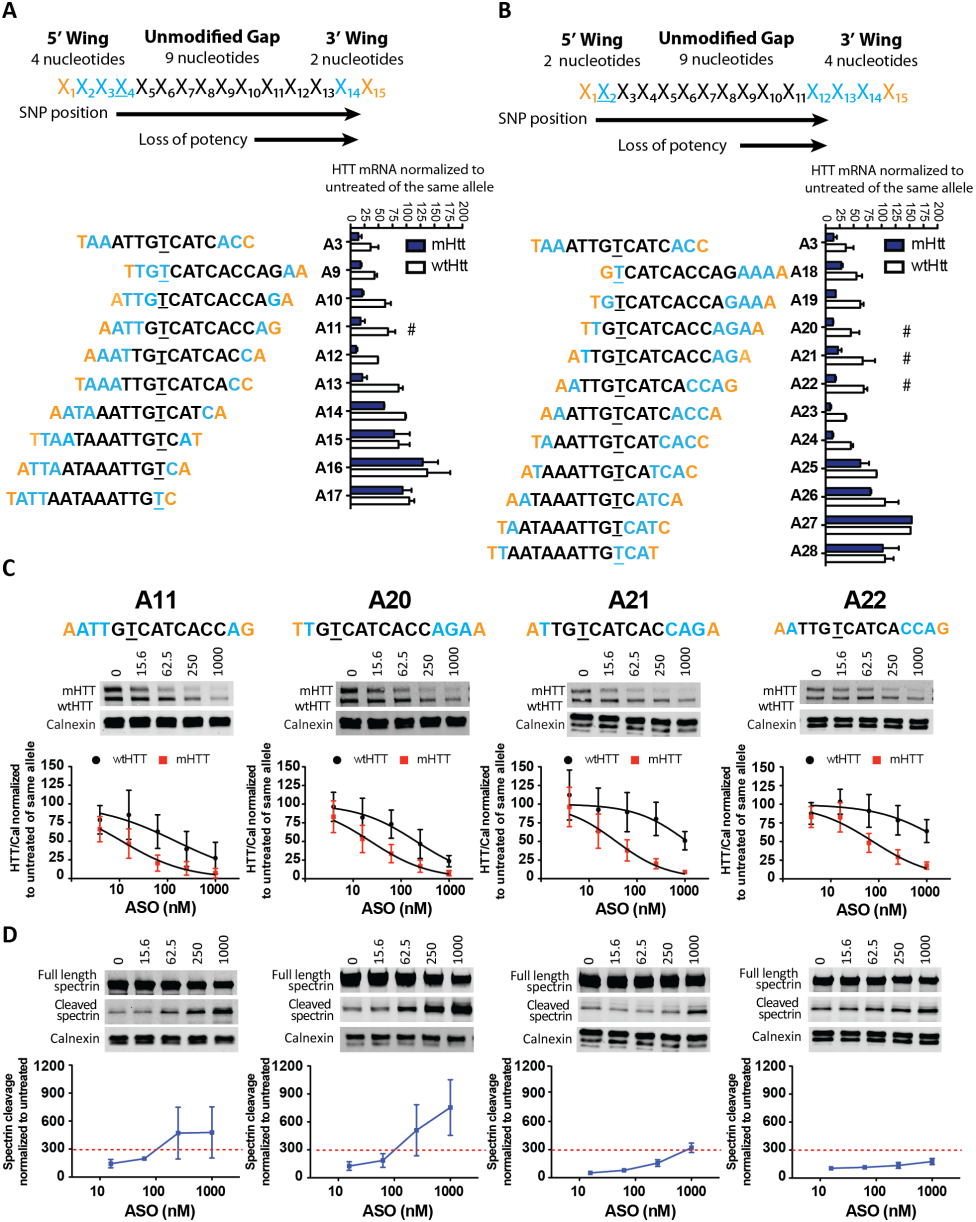
Microwalk of the SNP position within the gap. (A, B) Diagram of microwalk ASOs and HTT mRNA silencing in primary human HD fibroblasts. (A) Starting from A3, we moved one cEt modification to the 5′ wing (ekkk-9-ke) and moved the SNP site from position 4 to 14 (B) Similarly, we moved one cEt modification to the 3′ wing (ek-9-kkke) and moved the SNP site from position 2 to 12. mHTT and wtHTT mRNA were normalized to total RNA and then to the untreated sample. n = 2 per data point. A subset of ASOs from preliminary fibroblast screen marked by #, were evaluated in primary Hu97/18 neurons at 4–1000 nM for 6 days. (C) Western blots of HTT protein and quantitations. HTT levels are normalized to the internal loading control calnexin and then to the untreated sample for each allele. (D) Western blots showing full length and cleaved spectrin. Spectrin fragment is normalized to calnexin and then to the untreated sample. Membranes were probed for HTT and reprobed for spectrin. Representative images are shown. n = 6–10 per data point. Data are presented as mean ± SD. Two way ANOVA with Bonferroni post hoc test have been performed and p values are illustrated with *, **, ***, **** for p = 0.05, 0.01, 0.001, and 0.0001. The PS backbone is black, MOE and cEt modifications are illustrated by orange and blue, respectively. The SNP is underlined. The red dashed line represents the toxicity threshold.

We have previously demonstrated that RNase H cleaves to the 5′ -ASO/3′-RNA side of the SNP [34]. However, it is not completely clear whether the localization of the SNP position within the gap affects potency and specificity when it is moved towards either the 5′ or 3′ end of the molecule. This effect could presumably depend on the interaction between the ASO:RNA duplex and the RNase H enzyme. According to the crystal structure of RNase H, the enzyme makes extensive contact with the RNA:ASO heteroduplex at the 5′-RNA/3′-ASO side of the cleavage site on the RNA strand [57]. Therefore, we sought to determine if an asymmetrical wing design, providing higher affinity at either of the wings, could improve the ASO profile. First, using A3 as the parent molecule, we moved one cEt modification to the 5′ wing (ekkk-9-ke) and then in turn moved the SNP site from position 4 to 14 across the gap (Figure 4A). Similarly, we moved one cEt modification to the 3′ wing (ek-9-kkke) and then in turn moved the SNP site from position 2 to 12 across the gap of the ASO (Figure 4B). These 20 ASOs were first tested in a preliminary screen in primary human fibroblasts using a heterozygous cell line derived from an HD patient with the appropriate genotype at the relevant SNPs [33], [44]. The fibroblast cell line was treated at a single dose of 2 µM, and HTT mRNA suppression was evaluated using a SNP-based qPCR assay. We found a clear correlation between the position of the SNP and the potency of the ASO. Moving the SNP position towards the 3′ end of the gap resulted in loss of potency, whereas moving the SNP position towards the 5′ end of the gap maintained potency and specificity. This was consistent between both asymmetrical wing designs (Figure 4A and B and table S1).

To investigate these preliminary findings in more detail, we selected a subset of the ASOs with favourable properties, including A11, A20, A21, and A22, to be tested for potency, specificity, and toxicity in primary neurons (Figure 4C and D). Our aim was to identify ASOs with similar or better potency and greater specificity than our parent ASO, A3. The most active ASO, A23, showed better knock down of mHTT, but also greater knock down of wtHTT compared to A3, so it was not selected. A20 demonstrated the second greatest knock down of mHTT of the set and less knock down of wtHTT and was therefore chosen. The SNP positions for A21 and A22 were moved one nucleotide relative to A20. These oligos were marginally less potent, but slightly more specific and were selected for protein validation as well. A11 had an identical gap to the most promising ASO, A20, with the wing asymmetry reversed, and was therefore included to investigate the effect of wing chemistry. The four ASOs had IC_50_ values for mHTT from 11–78 nM, which is comparable to previously evaluated ASOs, suggesting that the number of modifications is more important than their distribution (Table 1). We did find an overall improvement in specificity for the four ASOs; ranging from 9 to more than 21 fold, suggesting that positioning the SNP nearer to the 5′ wing may be beneficial to specificity. However, since the motif of the chemical modifications is different from A3, the improvement may be a combination of the two factors. ASOs A11, A20, and A21 were excluded due to increased spectrin cleavage above threshold, whereas ASO A22 was well tolerated. ASO 22 showed potency in the upper end of the range (IC_50,mHTT_ = 78 nM) with robust specificity (>13 fold). However, at the highest dose of 1000 nM, A22 did cause a significant reduction in wtHTT expression of approximately 40%. Considering these data, the microwalk strategy did not provide sufficient improvement to specificity, and we therefore decided to move forward with investigation of shortening the gap of the oligo.

### Shortening the gap and length of the ASO

**Figure 5:**
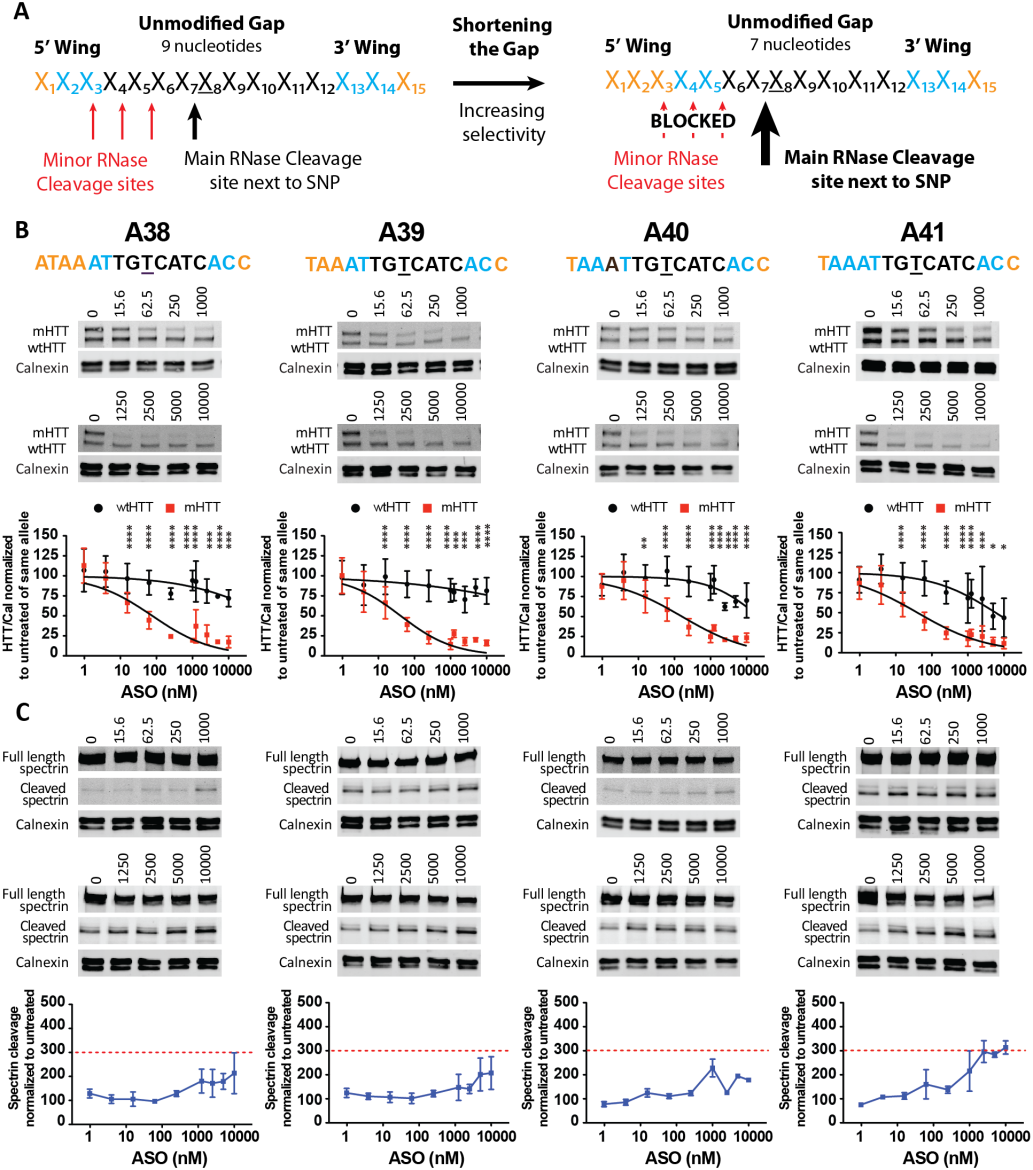
Shortening the gap to 7 nucleotides and evaluation at higher doses. (A) Replacing PS-nucleotides with RNase H resistant chemical modifications and shortening the gap from 9 to 7 nucleotides. The top 4 candidates are shown. Primary Hu97/18 neurons were treated with ASO at 1–10000 nM for 6 days. (B) Western blot and quantitation of HTT protein levels. HTT levels are normalized to the internal loading control calnexin and then to the untreated sample for each allele. (C) Western blots showing full length and cleaved spectrin. Spectrin fragment is normalized to calnexin and then to the untreated sample. Membranes were probed for HTT and reprobed for spectrin. Representative images are shown. n = 8–14 per data point at 0–1000 nM and n = 4–6 at 1250–10,000 nM. Data are presented as mean ± SD. Two way ANOVA with Bonferroni post hoc test have been performed and p values are illustrated with *, **, ***, **** for p = 0.05, 0.01, 0.001, and 0.0001. The PS backbone is black, MOE and cEt modifications are illustrated by orange and blue, respectively. The SNP is underlined. The red dashed line represents the toxicity threshold.

It is well described that RNase H cleaves within the sequence of the mRNA matching the gap of the ASO [58]. Therefore, the longer the gap, the more potential secondary sites are available for cleavage. Our group has previously demonstrated that shortening the gap of the ASO can increase specificity of mHTT mRNA knock down in human fibroblasts [34]. Here, we sought to validate these findings in a system that is more relevant to the brain by both evaluating protein knock down and toxicity after ASO treatment in primary neurons. Therefore, to increase specificity by preventing secondary cleavage events, we shortened the gap from 9 to 7 bases (Figure 5A) and synthesized a panel of 15-, 16-, and 17-oligomers (A29-A41) with different chemical wing motifs (Table 1). First, we tested A29 and A30, which have either five MOE or five cEt modifications in both wings, respectively. Exclusively using MOE modifications was not sufficient to achieve adequate suppression with a shorter oligo, whereas using full cEt wings resulted in high potency (IC_50,mHTT_ = 38 nM) and specificity (>26 fold). Unfortunately, A30 induced spectrin cleavage indicating that full cEt wings are not well tolerated for this specific sequence. Screening the remaining panel of ASOs, we found oligos with pronounced specificity (>56 fold) and high potency (IC_50_,_mHTT_ values as low as18 nM). However, the longer cEt modified ASOs (three out of five) were associated with toxicity, whereas the shorter oligos appeared more well tolerated with only one out of five inducing significant spectrin cleavage at the highest dose tested (Figure S8 and S9). Furthermore, the shorter oligos, including A38, A39, A40, and A41 showed minimal silencing of wtHTT across the doses (0–1000 nM) tested for the full panel of oligos (Figure 5B and Figure S8). Here, we confirm that by shortening the PS DNA gap, we can improve allele specificity without compromising potency or tolerability in a system pertinent to the brain.

Based on studies in non-human primates, it has become apparent that after intrathecal delivery, ASO concentration may differ significantly between areas close to or in direct contact with the cerebrospinal fluid, compared to the deeper structures of the brain [23]. Hence, it is fundamental to have a large therapeutic window, where the ASOs will be efficacious, non-toxic, and still remain specific for the mutant allele. Therefore, we wanted to determine the maximal dose of ASO that could be applied to primary neurons without overt toxicity and with minimal knock down of wtHTT. We treated primary neurons with our four lead ASO candidates at concentrations of up to 10,000 nM (Figure 5B). At the highest dose we observed spectrin cleavage just above threshold for ASO A41, whereas no spectrin cleavage above threshold was seen for ASOs A38, A39, and A40. Treatment with ASO A41 resulted in a 50% reduction of wtHTT at the highest dose used, whereas ASOs A38, A39 and A40 showed impressive specificity of 130, 147, and 60 fold, respectively, with only minimal reduction in wtHTT at extremely high doses of ASOs (Table 1). These findings demonstrate a great therapeutic window with more than 50% knock down of mHTT and a minimal effect on wtHTT levels over more than two log scale intervals.

**Figure 6:**
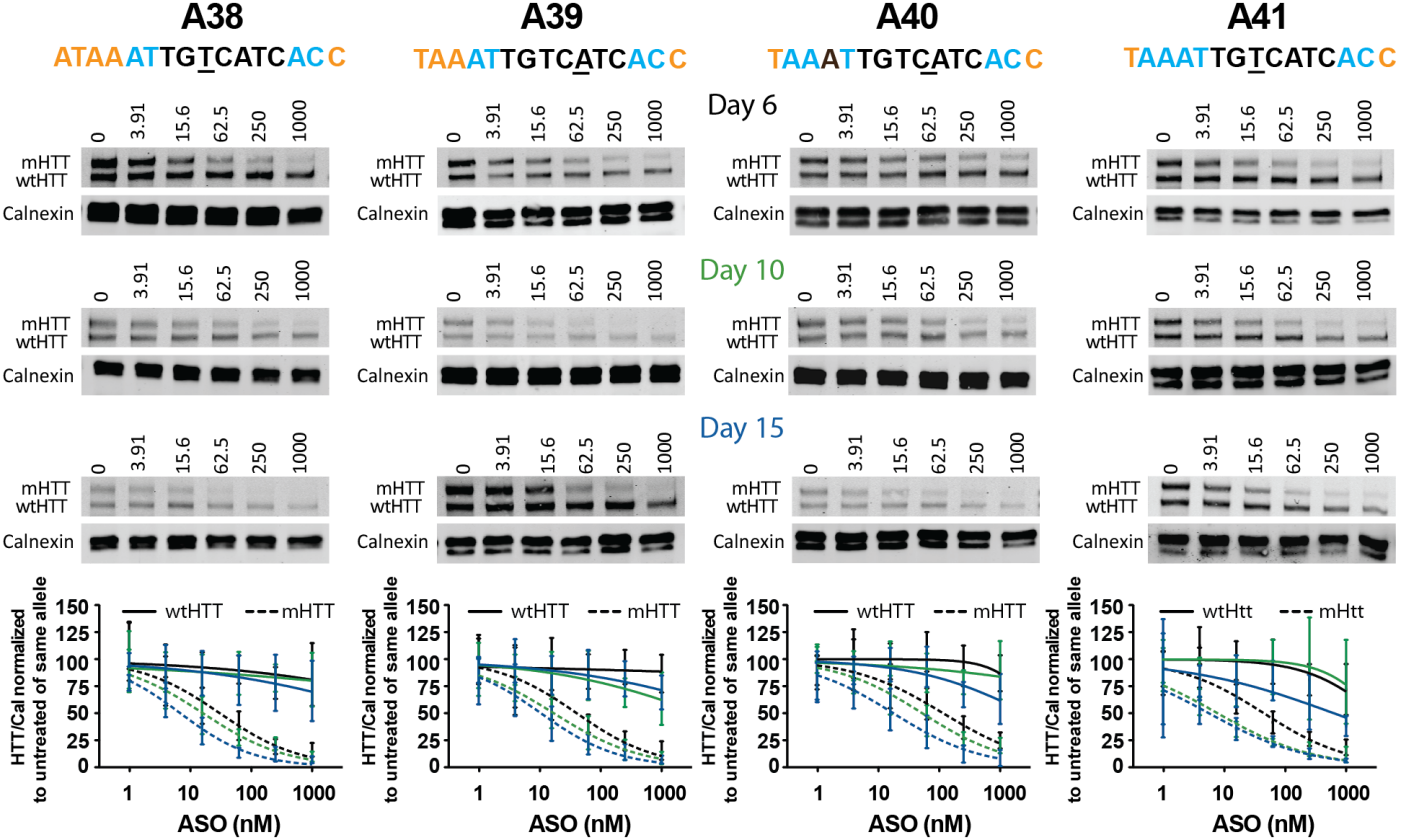
Increased potency with extended treatment duration. Primary Hu97/18 neurons were treated with ASO at 1–1000 nM for 6, 10, or 15 days. Western blot and quantitation of HTT protein levels. HTT levels are normalized to the internal loading control calnexin and then to the untreated sample for each allele. Representative images are shown. HTT protein levels (wtHTT = solid line, mHTT = dotted line) at day 6 (black), 10 (green), and 15 (blue). Data are presented as mean ± SD. n = 6–12 per data point. The IC_50_ values were compared using the extra-sum-of-squares F test and the F distribution and degrees of freedom F (DFn, DFd) and the associated p-values have been calculated. A38: day 6 vs. 10 F(1,106) = 7.254, P<0.0082; day 6 vs. 15 F(1,109) = 51.51, P<0.0001; day 10 vs. 15 F(1,99) = 18.88, P<0.0001; A39: IC_50,mHTT_ 38>17>10; day 6 vs. 10 F(1,115) = 13.94, P<0.0003; day 6 vs. 15 F(1,98) = 25.06, P<0.0001; day 10 vs. 15 F(1,21) = 5.625, P<0.0193); A40: IC_50,mHTT_ 122>53>18, day 6 vs. 10 F(1,67) = 6.030, P<0.0167; day 6 vs. 15 F(1,58) = 30.25, P<0.0001; day 10 vs. 15 F(1,61) = 12.68, P<0.0007); A41: IC_50,mHTT_ 45>8>6; day 6 vs. 10 F(1,85) = 66.19, P<0.0001; day 6 vs. 15 F(1,76) = 47.82, P<0.0001; day 10 vs. 15 F(1,79) = 1.258, P<0.2655). The PS backbone is black, MOE and cEt modifications are illustrated by orange and blue, respectively. The SNP is underlined.

Since ASOs have a relatively long tissue half-life [59], it is important that specificity is maintained over time. To investigate this, we extended the treatment duration from 6 days to 10 and 15 days. As expected with longer treatment duration, increased suppression of mHTT was observed for all ASOs tested. Non-linear regression demonstrates that IC_50_ values for lowering of mHTT decrease with longer treatment durations (A38; IC_50,mHTT_ 32>16>7; A39; IC_50,mHTT_ 38>17>10; A40; A41; IC_50,mHTT_ 45>8>6). Despite increased activity, specificity of mHTT silencing was maintained over increased treatment durations for 3 of 4 leads. ASOs A38, A39, and A40 showed minimal silencing of wtHTT, whereas there was greater reduction in wtHTT levels after longer treatments with A41 (Figure 6 and Table 1).

**Figure 7:**
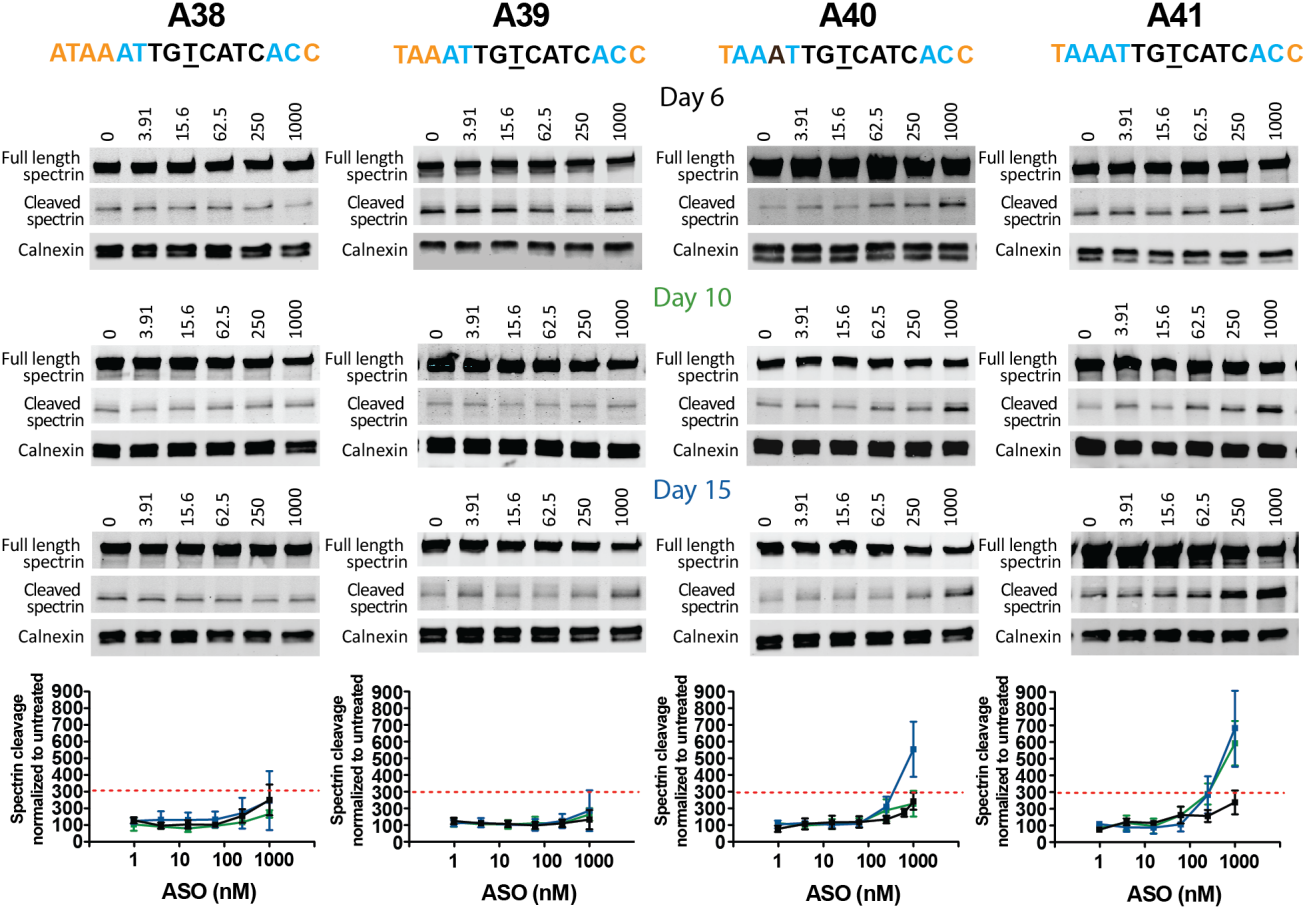
Spectrin cleavage after extended treatment duration. Primary Hu97/18 neurons were treated with ASO at 1–1000 nM for 6, 10, or 15 days. Western blots showing full length spectrin and cleaved spectrin (120 kDa) after 6 (black), 10 (green), and 15 (blue) days of treatment. Spectrin fragment is normalized to calnexin and then to the untreated sample. HTT membranes were reprobed for spectrin. Representative images are shown. Data are presented as mean ± SD with n = 6–12 per data point. The PS backbone is black, MOE and cEt modifications are illustrated by orange and blue, respectively. The SNP is underlined. The red dashed line represents the toxicity threshold.

To further improve the sensitivity of our triage, we wanted to explore if longer treatment durations would reveal subtle differences in tolerability. We observed increased cleavage of spectrin after 10 days of treatment with ASO A41 and after 15 days of treatment with either A40 or A41 (Figure 7), indicating that these two ASOs are not well tolerated over long treatment durations. We did not observe cleavage of spectrin above threshold for A38 and A39 after the extended treatment durations. These comprehensive analyses allowed us to characterize subtle differences between the four candidate ASOs and identify ASOs A38 and A39 as the most promising leads.

### Targeting both alleles at a single HD-SNP could provide a therapy to all HD patients

The steps described here are the initial process towards the construction of a panel of ASOs to provide allele-specific silencing to the majority of HD patients. However, it will take time to achieve this goal and meanwhile all therapeutic options should be considered for the remaining HD patients until this panel is established. We have previously observed that 10.7% (7 out of 65) of HD patients are homozygous at 22 genotyped SNPs [44] and would not be treatable allele-specifically with ASOs targeted to those sites. To further investigate and substantiate these findings, we have analysed genotypes from an expanded panel of 91 SNPs [33], and similarly find that 11.5% (27 out of 234) of patients are homozygous at the SNPs tested in this assay. These data illustrate the need for an alternative approach for this group until additional allele-specific targets may be identified.

**Figure 8:**
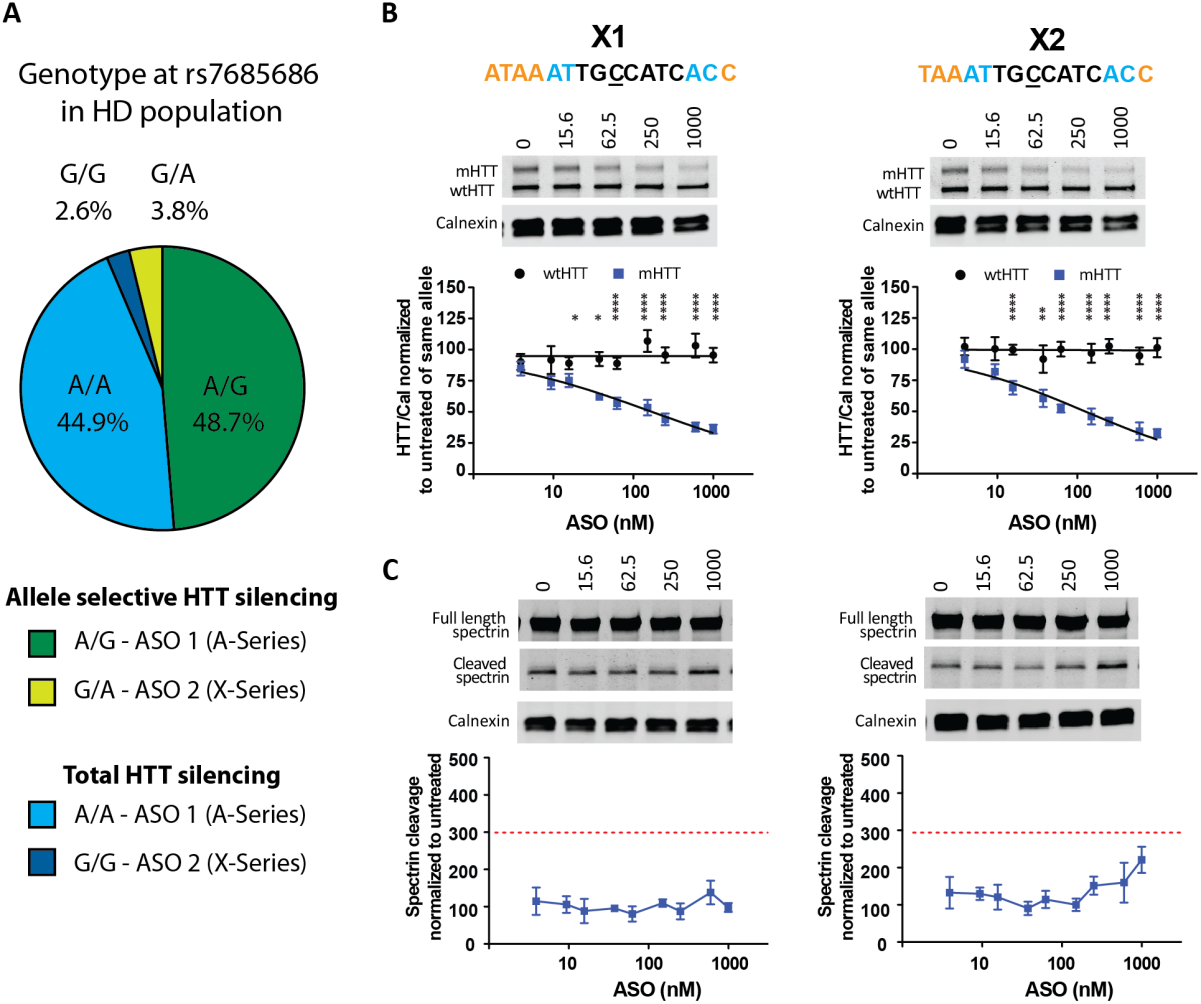
Targeting two variants of a single HD-SNP to provide a therapeutic option to all HD patients. (A) The genotypes for the sequenced HD population at rs7685686. Green = heterozygous HD population (rs7685686_A/G, 48.7%, targetable by A-series ASOs and rs7685686_G/A, 3.8%, targetable by X-series ASOs). Blue = homozygous HD population (rs7685686_A/A, 44.9%, targetable by A-series ASOs and rs7685686_G/G, 2.6%, targetable by X-series ASOs). Primary YAC128 neurons were treated with ASO at 16–1000 nM for 6 days. (B) Western blot and quantitation of HTT protein levels. HTT levels are normalized to the internal loading control calnexin and then to the untreated sample for each allele. (C) Western blots showing full length and cleaved spectrin. Spectrin fragment is normalized to calnexin and then to the untreated sample. Membranes were probed for HTT and reprobed for spectrin. Representative images are shown. n = 8–12 per data point. Data are presented as mean ± SD. Two way ANOVA with Bonferroni post hoc test have been performed and p values are illustrated with *, **, ***, **** for p = 0.05, 0.01, 0.001, and 0.0001. The PS backbone is represented by black. MOE and cEt modifications are illustrated by orange and blue, respectively. The SNP is underlined. The red dashed line represents the toxicity threshold.

Our lead ASO candidates such as A38 or A39 that target rs7685686_A, could provide an allele-specific therapeutic option for 48.7% of the sequenced HD population [33]. Using our custom SNP genotyping assay data, we show that 44.9% of HD patients are homozygous at this SNP having an adenine on both alleles (rs7685686_A/A) (Figure 8A). Therefore, our ASOs targeting rs7685686_A could potentially provide a treatment option for a total of 93.6% of all HD patients, where approximately half would be allele-specific and the other half would be non-allele specific. Among the remaining 6.4% of the HD population, we find that 3.8% are heterozygous, with a guanine on the mutant allele and an adenine on the wt allele (rs7685686_G/A), and 2.6% are homozygous with a guanine on both alleles (rs7685686_G/G). Our lead ASOs targeting the adenine allele would not provide a therapeutic option for this minority of patients. Therefore, we investigated if ASOs analogous to A38 and A39 but having thymine exchanged for cytosine at the SNP position would be active against rs7685686_G (Figure 8B). To screen these oligos in an appropriate system, we used primary neurons from YAC128 mice, which carry a mutant human transgene with the guanine genotype at rs7685686 and endogenous murine Hdh gene. Because the endogenous murine Hdh genes do not share any sequence similarity to human HTT around this SNP site, we were unable to evaluate specificity and instead focused on potency and tolerability. As previously, neurons were treated with ASOs for 6 days and protein was collected for analysis. We found increased knock down of mHTT with increasing dose of ASO and, as expected, no change in the levels of endogenous murine Htt (Figure 8B). Similar to their analogs, ASOs X1 and X2 did not induce spectrin cleavage above threshold (Figure 8C). However, ASO X1 and X2 had slightly higher IC_50_ values for mHTT (150 and 134 nM, respectively) than was observed for A38 and A39, which demonstrates the impact of changing one of the 15 or 16 nucleotides in the oligo (Table 1). These ASOs provide an excellent starting point for additional SAR studies to identify ASOs targeting rs7685686_G with properties similar to ASOs A38 and A39.

## Discussion

We have established a pipeline that enables us to assess the ASO activity at multiple SNP targets and further discriminate between safe and toxic oligos in a system relevant to the brain. We have identified lead ASO candidates for in vivo validation and demonstrated that targeting two allelic variants of a single HD-SNP can be used as a therapeutic option, either allele-specific or non-specific, for all carriers of the HD mutation, using two distinct ASO drugs till additional allele-specific SNPs and supplementary ASOs are identified and developed.

### Screening pipeline

Primary neurons with the appropriate genetic background including human transgenic wt and mutant HTT and without the presence of endogenous murine Htt are an ideal system for rapid *in vitro* screening of gene silencing drugs for the brain. The use of primary neurons allow us to screen for the potency and allele-specificity of a large number of ASO modifications against a great number of SNP targets, and test a wide range of ASO concentrations (0–10,000 nM), which is one to two orders of magnitude higher than other current screening systems [27], [36], [60], [61]. Furthermore, this system provides a sensitive way to exclude toxic ASOs before they go into pre-clinical animal studies resulting in increased efficiency and reduced research costs. Providing availability of genetically appropriate mouse models, this screening approach would be amendable to other dominant monogenetic neurological disorders and can be adapted for screening ASOs, RNAi or other SNP based therapies.

### ASO design

Our data demonstrate that initially selecting multiple sites for evaluation is critical, since ASOs at all SNP sites are not equally active. This is most likely caused by secondary and tertiary RNA structures that can either prevent binding of the oligo to the target RNA or sterically hinder the recruitment of the RNase-H enzyme to the ASO:RNA duplex [62], [63]. After identifying the SNP sites (rs7685686, rs4690072, rs2024115, rs363088) where the ASOs show the most activity, we have evaluated several ASO design strategies to facilitate potent and specific silencing of mHTT. We find that the incorporation of cEt-modified nucleotides dramatically improves potency (e.g. A29 vs. A30). However, we have not been able to clearly establish a consensus motif for wing modification that is superior to others tested. Similarly, we have not clearly isolated the individual factors that affect the safety profile of the ASO, which are comprised of multiple elements including the target, the length and sequence, and the modification motif of the wings. However, we have established that shorter oligos are generally better tolerated. We have corroborated that shortening the gap region increases specificity dramatically by decreasing the number of potential secondary RNase cleavage sites. Furthermore, our investigations have shown that there is some flexibility for the SNP position within the gap of the ASO. The SNP can be moved from the center towards the 5′ wing while maintaining potency and specificity, which allows for microwalking and identification of ASOs with a potentially better tolerability profile.

After improving the ASO design and incorporating cEt modifications in combination with MOE chemistry, we find the potency of our ASOs to be in the lower nanomolar range comparable to what has been observed in other *in vitro* systems using SiRNA, LNA oligos, single-stranded RNA, unmodified or modified RNA duplexes [27], [28], [36], [37], [61]. However, a direct comparison is not completely possible, since the actual intracellular concentration of drug will depend on delivery method e.g. free uptake versus transfection or electroporation. Furthermore, the potency will be contingent on the treatment duration and whether protein or RNA are used as a readout. Similarly, these variables in addition to the maximal concentration of drug being used may also affect the calculated specificity. Several research groups have shown promising results targeting the CAG expansion in a cell line from a juvenile HD patient (CAG 69/17) with specificity ranging from 30–71 fold. However, when using these drugs in cell lines with CAG expansions that are more representative of the general HD population (CAG 44/15, 44/21, and 47/18), specificity decreases, and there is loss of close to 50% of wtHTT expression [27], [38], [42]. Østergaard et al. have previously shown great specificity of >133 fold at the RNA level when targeting HD-SNPs in fibroblasts. In this study, we have found specificity of >147 fold at the protein level in primary neurons with negligible effect on wtHTT levels, which is a substantial improvement compared to most previously published studies for both SNP-targeted as well as CAG-targeted approaches suppressing mHTT protein expression [30]–[32], [34]. Importantly, these findings are achieved without any carrier or delivery vehicle, since the ASOs are freely taken up by the neurons. We have developed two very strong lead ASOs, with low nanomolar IC_50_ values by free uptake into primary neuronal cells and impressive specificity, against rs7685686_A suitable for *in vivo* validation. Furthermore, our findings provide some insight into advantageous oligo design that can be used as a starting point for sequential screening of secondary and tertiary ASO candidates.

### A therapeutic option to all HD patients

The steps described here are the initial process towards the long term goal of constructing a panel of ASOs to provide allele-specific silencing to all HD patients. We are currently in the process of re-populating our ASO pipeline using relevant HD-SNP targets that will add additional patient coverage. We believe that screening at these complementary sites will be faster and more efficient using information garnered from this screen. Despite this increased efficiency, building a full panel of allele-specific ASOs will take significant time. Another concern that has been raised is that some people with HD may not currently be targetable with this approach. Previous genetic population studies indicate that a minority of HD patients are homozygous at all investigated HD-SNPs. Warby et al. explored a panel of 22 SNPs and found that 7 out of 67 HD patients were homozygous at these SNPs [44]. Similarly, Pfister et al. assessed 22 SNPs (18 differed from the Warby panel) in 109 patients and found that the maximal percentage of patients with at least one heterozygous SNP reached a plateau at approximately 80% [32]. This study does not provide the actual number of homozygous patients, but it can be inferred that about a fifth of patients in this study are homozygous at the 22 genotyped SNPs. To substantiate these findings, we analysed an expanded panel of 91 SNPs in 234 patients and found that 11.5% are homozygous at the 91 SNPs in this panel [33]. These findings taken together demonstrate that we need to identify novel HD-SNPs to provide an allele-specific therapeutic option to the group of patients that are homozygous at all assayed SNPs. During the time it takes to define and validate new targets and develop new ASOs, alternative strategies have to be employed to provide the best outcome for all patients and to make sure that some therapeutic options is available to all patients. As previously mentioned, there are concerns with non-specific HTT knock down, as we cannot fully comprehend the consequences of loss of wtHTT function in the adult human brain over longer terms. However, if intermittent or short term non-specific ASO treatment could provide benefit for HD patients during the development of complementary allele-specific ASOs, it would be worth considering.

As a start, our lead ASOs targeting rs7685686_A, could provide an allele-specific therapeutic option for 48.7% of HD patients. In addition, they could provide a non-specific HTT silencing option for 44.9% of HD patients that are homozygous (rs7685686_A/A). This means that one of our lead ASOs could potentially provide a therapeutic option to 93.6% of people with HD. Since, we have found that rs7685686 is an accessible SNP site, we have explored the possibility of targeting the opposite allele at the same SNP site (e.g. ‘G’ vs. ‘A’) to provide a therapeutic option for the remaining 6.4% of patients. Targeting rs7685686_G would provide an allele-specific therapeutic option to 3.8% and a non-allele-specific option to 2.6% of HD patients.

With this strategy in mind, we designed two ASOs, X1 and X2, that are analogous to our leads, A38 and A39, and evaluated them in primary neurons from YAC128 mice. ASOs X1 and X2 showed good activity (IC_50_,_mHTT_ of 150 and 134 nM) and were well tolerated in our screens. Overall, these findings show that two ASOs targeted to the two allelic variants of a single SNP could provide a therapeutic option for all HD patients, where roughly half would receive an allele-specific therapy and the remaining patients would receive a non-specific therapy. This strategy could potentially provide benefit during the time it takes to develop a complete allele-specific ASO panel. While there are safety concerns for long-term reduction of wtHTT, in short term, a non-specific HTT silencing therapy would likely be preferable to untreated HD.

### Translation of *in vitro* ASO screen

We have previously demonstrated that our *in vitro* findings translate well to the brains of transgenic mice [33], [34]. Here we show that our lead oligos, A38 and A39, induce robust suppression of mHTT while maintaining great specificity over more than two log scale intervals (100–10,000 nM). This large therapeutic window will be essential for successful *in vivo* efficacy and tolerability studies, since it has become apparent that therapeutic doses of ASOs delivered via the cerebrospinal fluid to the brain result in a concentration gradient of ASO across the non-human primate brain [23], [64]. Ideally, the lower concentration of ASO in the deeper areas of the brains would be sufficient for mHTT suppression, whereas higher amounts of ASOs in the outer areas of the brain would suppress mHTT without affecting wtHTT levels or inducing toxicity.

Analogous to other drugs, ASOs have the risk of causing unintended toxicity, which may result from three different mechanisms; the reduction of the target to an extent that leads to adverse outcomes, hybridisation independent events such as nucleic acid-protein interactions, and/or hybridisation-dependent events such as binding to unrelated RNA targets [65]. Currently, there are no algorithms to predict these events and each ASO has to be fully evaluated independently for safety through *in vivo* studies in animals and subsequently in carefully controlled human clinical trials [65]. Contingent on pre-clinical validation, the translation into analogous human clinical studies could be rapid, especially considering the latest ASO trials. The first human clinical trial using antisense therapy for a neurodegenerative disease was completed last year for amyotrophic-lateral-sclerosis using intrathecal delivery of ASO. No safety or tolerability concerns were found [48]. Similarly, no safety issues have been reported for an ongoing spinal muscular atrophy trial using intrathecal injection of ASO (ClinicalTrials.gov Identifier: NCT01494701). So far, two ASO drugs have been approved by the FDA, fomivirsen, given intraocularly, and mipomersen, given systemically, and numerous others currently in clincal trials [46], [66]. Since the first initial experiments with ASOs targeting HTT more than a decade ago, antisense technologies have come a long way and we are entering a new era of gene silencing. The path from ASO development to the clinic is steadly becoming more feasible with increasing knowledge.

## Materials and Methods

### Genotyping of patient material

We have previously designed a genotyping panel of 96 SNPs using a Goldengate assay on the Illumina BeadArray platform [33]. Briefly, 96 SNPs were selected for the genotyping assay based on LD patterns from Hapmap, dbSNP and in-house sequencing. DNA samples from the Huntington Disease BioBank at the University of British Columbia from 390 different HD pedigrees were collected. 1151 samples were genotyped using Illumina GenomeStudio v2011 and subsequently phased based on information from family trios using the PHASE 2.0 software.

### Ethics statement

Consent and access procedures were in accordance with institutional ethics approval for human research (UBC certificate H05-70532). Publically available human fibroblasts cell lines were obtained from NIGMS Human Genetic Cell Repository at the Coriell Institute for Medical Research (http://ccr.coriell.org). Animal experiments were performed with the approval of the animal care committee at the University of British Columbia.

### Fibroblasts

Fibroblast line GM04022, which is heterozygous at SNP rs7685686_A, was used to measure the *in vitro* potency of the modified ASOs at the mRNA level according to previous protocols [34]. In short, the cells were transfected with 2 µM ASO (or 3 µM for ASOs A15 and A21) by electroporation (Harvard Apparatus ECM830, 115 V, 6 msec) and RNA was extracted 24 h later using the Qiagen RNeasy96 kit according to the manufacturer’s specifications. Expression of human *HTT* mRNA alleles was quantified using a qPCR assay at SNP rs362331 (C_2231945_10, Life Technologies). Quantitative RT-PCR reactions were run on the ABI 7900HT instrument using the Quantitect Probe RT-PCR kit following the manufacturer’s instructions. Total RNA content measured by Ribogreen was used for normalization.

### Mice and breeding

Mice were housed under a 12 hour light and dark cycle in a clean facility with free access to food and water. Hu18/18 and Hu97/18 [51] timed matings were established, producing offspring of 50% each genotype. Hu97/18 embryos were used to set up primary neuronal cultures. YAC128 (line 53) [67] mice were crossed with FVB mice, and the transgene positive embryos were used for neuronal cultures.

### Genotyping of mice

Embryos were collected on day 15.5–16.5 of gestation. Brains were extracted and transferred to Hibernate E (Invitrogen) for 24 hrs, allowing maintenance of neuron viability until genotyping was completed. Tail tissue for genotyping was collected from each embryo, and DNA was extracted using the QuickLyse Miniprep Kit (Qiagen). For Hu97/18 embryos, a PCR across the CAG expansion was used to distinguish between the two human HTT transgenes (forward primer: 5′ - ATTGCCCCGGTGCTGAGCG -3′ and reverse primer: 5′ - GCGGGCCCAAACTCACGGTC-3′) yielding product sizes of 351bp and 588bp for the YAC18 and BACHD alleles, respectively. For the YAC128 embryos, two PCRs at each of the YAC arms were used to confirm the presence of the full YAC insert. Actin was used as positive PCR control. Left YAC arm PCR (forward primer: 5′ - CCTGCTCGCTTCGCTACTTGGAGC-3′ and reverse primer: 5′ - GTCTTG CGCCTTAAACCAACTTGG-3′) yielding a product size of 230bp. Right YAC arm PCR (forward primer: 5′ - CTTGAGATCGGGCGTTCGACTCGC-3′ and reverse primer: 5′ - GTCTTGCCGCACCTGTGGCGCCGGTGATGC-3′) yielding product size of 170bp. Actin PCR (forward primer: 5′ - AGCCTCAGGGCATCGGAACC-3′ and reverse primer: 5′ - GGAGACGGGGTCACCCACAC-3′) yielding product size of 450bp.

### Primary neuronal culture and ASO treatment

Embryonic brains were removed from Hibernate E, and the forebrains microdissected in ice-cold Hank’s Balanced Salt Solution (HBSS+; Gibco) to remove the hippocampi, isolating the cortex and striatum, which was used to set up neuronal cultures. The tissue was minced and digested with 0.05% Trypsin-EDTA (Invitrogen) at 37°C for 8 minutes, and trypsin was subsequently neutralized with 10% Fetal Calf Serum (FCS; Gibco) in Neuro Basal Medium (NBM; Gibco). Cells were resuspended in complete culture media (NBM+), NBM containing 2% B27 (Gibco), 100 U/ml PS, and 0.5 mM L-Glutamine (Gibco), and treated with DNAse I (153 U/µl) (Invitrogen). Tissue was triturated 5–6 times with a 5 ml serological pipette, and cells were counted and seeded at 1.2×10^6^ cells/well on poly-D-lysine coated 6-well plates in 2 ml of NBM+. Primary neuronal cultures were maintained in a humidified incubator at 37°C and 5% CO_2_. Neurons were treated with 200 µl ASOs in fresh medium on the second day *in vitro* (DIV) and fed with 200 µl fresh medium every fifth day post treatment. Images were taken with EVOS XL Core Imaging System from Life Technologies with a 10X objective. Size marker was added to the images using a calibration grid slide (250 uM grids) from MBF Bioscience. As a positive control for spectrin cleavage, we used camptothecin, a topoisomerase inhibitor, to induce apoptosis. At DIV8 increasing concentrations of campthothecin were added to Hu97/18 neurons and spectrin cleavage was evaluated after 24 hours of stress.

### Western blotting

Cortical and striatal neurons were collected from the culture dish on DIV 8, 12, or 17 by scraping in ice cold PBS and pelleting by centrifugation at 2400 g for 5 min at 4°C. Dry pellets were then stored at −80°C. Proteins were extracted by lysis with SDP+ buffer and 20–40 µg of total protein was resolved on 10% low-BIS acrylamide gels and transferred to 0.45 µm nitrocellulose membrane as previously described [33]. Membranes were blocked with 5% milk in PBS, and then blotted with the anti-HTT antibody 2166 (Millipore) for detection of HTT. Anti-calnexin (Sigma C4731) immunoblotting was used as a loading control. Membranes were scanned and HTT and Calnexin levels were quantified. Subsequently, the membranes were reprobed with anti-spectrin antibody (Enzo BML-FG6090) and the caspase-3 cleaved 120-kDa fragment of alpha-II-spectrin was quantified. Spectrin cleavage was used as a readout for apoptosis induction to evaluate toxicity of each ASO. Representative images for HTT and spectrin were chosen to best match the data. Proteins were detected with IR dye 800 CW goat anti-mouse (Rockland 610-131-007) and AlexaFluor 680 goat anti-rabbit (Molecular Probes A21076)-labeled secondary antibodies using the LiCor Odyssey Infrared Imaging system. Licor Image Studie Lite was used to quantify the intensity of the individual bands.

### Data analysis

Data are expressed as means±SD. Results were analysed using non-linear regression with normalized response and a variable curve. The IC_50_ values were compared using the extra-sum-of-squares F test and the F distribution and degrees of freedom F (DFn, DFd) and the associated p-values have been calculated. Allele specificity was calculated by dividing the IC_50_ for wtHTT by the IC_50_ for mHTT. If the IC_50_ for reducing wtHTT was greater than the highest ASO concentration tested, then allele specificity was calculated by dividing the highest ASO concentration tested by the IC_50_ for mHTT reduction and expressed as >fold. Two way ANOVA with Bonferroni post hoc test have been performed to determine if mHTT expression is different from wtHTT levels at each individual dose of oligo tested. Analyses were performed using GraphPad Prism Ver.5. Differences were considered statistically significant when p<0.05.

## Supporting Information

Figure S1 **Spectrin cleavage assay.** To enable a successful triage and exclusion of toxic ASOs, we measured the level of the 120 kDa spectrin cleavage fragment normalized to calnexin loading control, and then to the untreated sample. Camptothecin induced spectrin cleavage was used as a positive control. Representative Western blots and spectrin quantification from a non-toxic and a toxic ASO are shown. n = 4–6 per data point. Data is presented as mean ± SD. The red dashed line represents the toxicity threshold.
(TIF)

Figure S2 **Selection of favourable SNP targets – HTT levels.** Hu97/18 neurons were treated with 5e-9-5e ASOs targeted to 10 HD-SNPs and HTT protein level was analyzed. HTT levels were normalized to calnexin and then to the untreated sample for each allele. Representative images are shown. n = 4–6 per data point. Data are presented as mean ± SD. The PS backbone is represented by black; MOE modifications are illustrated by orange. The SNP is illustrated by the underlined nucleotide.
(TIF)

Figure S3 **Selection of favourable SNP targets – Spectrin cleavage.** Hu97/18 neurons were treated with 5e-9-5e ASOs targeted to 10 HD-SNPs and spectrin cleavage was analyzed. The 120 kDa fragment was normalized to calnexin and then to the untreated sample. HTT membranes were reprobed for spectrin. Representative images are shown. n = 4–6 per data point. Data are presented as mean ± SD. The # denotes two ASOs that induced rearrangement of the neurons. The PS backbone is represented by black; MOE modifications are illustrated by orange. The SNP is illustrated by the underlined nucleotide. The red dashed line represents the toxicity threshold.
(TIF)

Figure S4 **Altered neuronal morphology after treatment with some ASOs.** Treatment with ASOs E1 and G1 caused marked morphological changes at the highest doses tested (1000 nM) resulting in rearrangement of neuronal cell bodies into an organized network. Representative images are shown of treated and untreated neurons. Black arrows indicate cell bodies grouped together connecting to other cell clusters. Images were taken with EVOS XL Core Imaging System from Life Technologies using the 10X objective. A calibration grid slide with 250 uM grids from MBF Bioscience was used to add a size marker to the images.
(TIF)

Figure S5 **Targeting 4 SNPs using two different cEt motifs – HTT levels.** Hu97/18 neurons were treated with ASOs with cEt modified wings and HTT protein was analyzed. HTT levels were normalized to calnexin and then to the untreated sample for each allele. Representative images are shown. n = 6–10 per data point. Data are presented as mean ± SD. The PS backbone is represented by black; MOE and cEt modifications are illustrated by orange and blue, respectively. The SNP is illustrated by the underlined nucleotide.
(TIF)

Figure S6 **ASO screen at 4 SNPs using two different cEt motifs – Spectrin.** Hu97/18 neurons were treated with ASO with cEt modified wings and spectrin cleavage was analyzed. The 120 kDa fragment was normalized to calnexin and then to the untreated sample. HTT membranes were reprobed for spectrin. Representative images are shown. n = 6–8 per data point. Data are presented as mean ± SD. The PS backbone is represented by black; MOE and cEt modifications are illustrated by orange and blue, respectively. The SNP is illustrated by the underlined nucleotide. The red dashed line represents the toxicity threshold.
(TIF)

Figure S7 **Wing SAR study.** Hu97/18 neurons were treated with ASO with cEt modified wings and HTT protein and spectrin cleavage was analyzed. HTT levels were normalized to calnexin and then to the untreated sample for each allele. The 120 kDa fragment was normalized to calnexin and then to the untreated sample. Membranes were probed for HTT and reprobed for spectrin. Representative images are shown. n = 4–6 per data point. Data are presented as mean ± SD. The PS backbone is represented by black; MOE and cEt modifications are illustrated by orange and blue, respectively. The SNP is illustrated by the underlined nucleotide. The red dashed line represents the toxicity threshold.
(TIF)

Figure S8 **Shortening the gap to 7 nucleotides – HTT levels.** Replacing PS-nucleotides with RNase H resistant nucleotides and shortening the gap increases selectivity by preventing cleavage at secondary cleavage sites and restricting cleavage to the main site next to the targeted SNP. Hu97/18 neurons were treated with ASOs and HTT protein was analyzed. HTT levels were normalized to calnexin and then to the untreated sample for each allele. Representative images are shown. n = 6–10 per data point. Data are presented as mean ± SD. The PS backbone is represented by black; MOE and cEt modifications are illustrated by orange and blue, respectively. The SNP is illustrated by the underlined nucleotide.
(TIF)

Figure S9 **Shortening the gap to 7 nucleotides – Spectrin cleavage.** Hu97/18 neurons were treated with ASOs and spectrin cleavage was analyzed. The 120 kDa fragment is normalized to calnexin and then to the untreated sample. HTT membranes were reprobed for spectrin. Representative images are shown. n = 6–8 per data point. Data are presented as mean ± SD. The PS backbone is represented by black; MOE and cEt modifications are illustrated by orange and blue, respectively. The SNP is illustrated by the underlined nucleotide. The red dashed line represents the toxicity threshold.
(TIF)

Table S1 **Summary of ASO RNA screen in human fibroblasts.** MOE and cEt modifications are annotated by e and k, respectively. The SNP is underlined.
(DOCX)
