# Supplementary figures and images for: Allele-Specific Suppression of Mutant Huntingtin Using Antisense Oligonucleotides: Providing a Therapeutic Option for All Huntington Disease Patients
Source: PLoS One. 2014 Sep 10;9(9):e107434. doi: 10.1371/journal.pone.0107434 (PMC4160241; doi:10.1371/journal.pone.0107434)

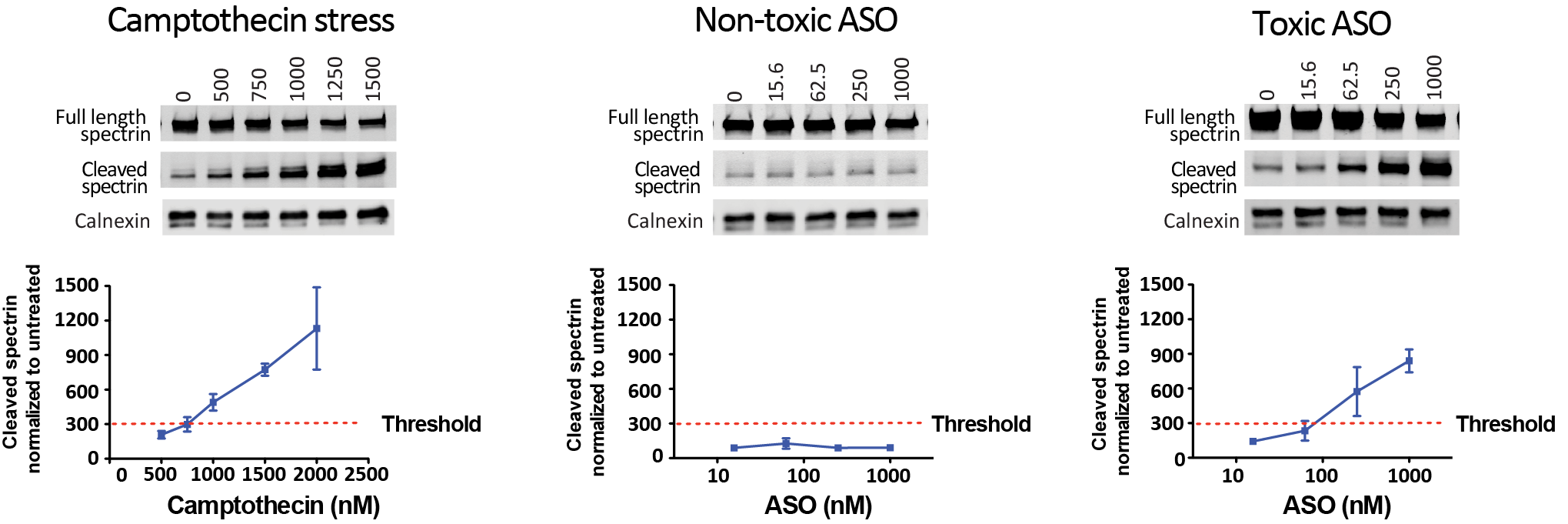

Supplement: Figure S1 — Spectrin cleavage assay. To enable a successful triage and exclusion of toxic ASOs, we measured the level of the 120 kDa spectrin cleavage fragment normalized to calnexin loading control, and then to the untreated sample. Camptothecin induced spectrin cleavage was used as a positive control. Representative Western blots and spectrin quantification from a non-toxic and a toxic ASO are shown. n = 4–6 per data point. Data is presented as mean ± SD. The red dashed line represents the toxicity threshold. (TIF) [file pone.0107434.s001.tif]

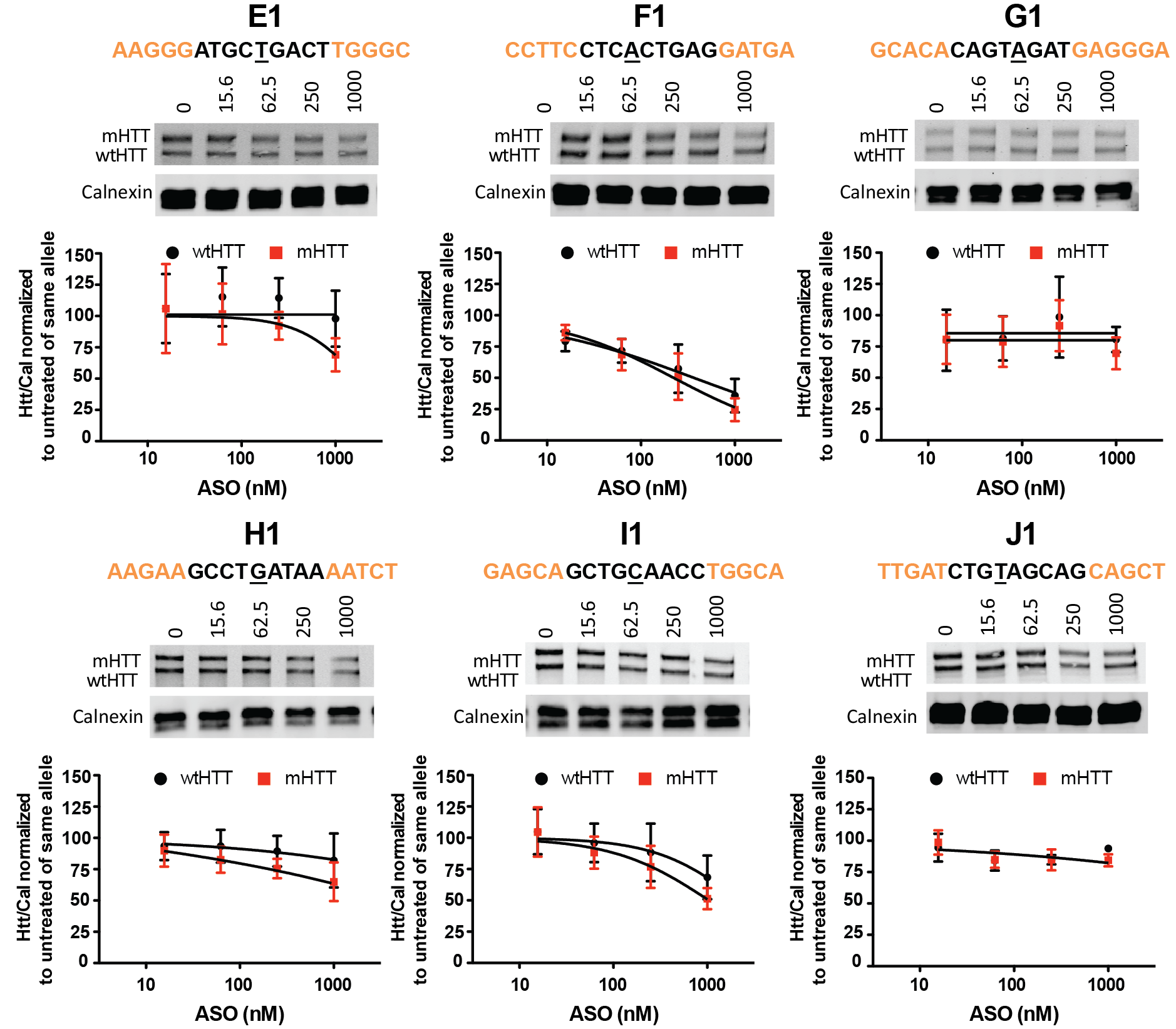

Supplement: Figure S2 — Selection of favourable SNP targets – HTT levels. Hu97/18 neurons were treated with 5e-9-5e ASOs targeted to 10 HD-SNPs and HTT protein level was analyzed. HTT levels were normalized to calnexin and then to the untreated sample for each allele. Representative images are shown. n = 4–6 per data point. Data are presented as mean ± SD. The PS backbone is represented by black; MOE modifications are illustrated by orange. The SNP is illustrated by the underlined nucleotide. (TIF) [file pone.0107434.s002.tif]

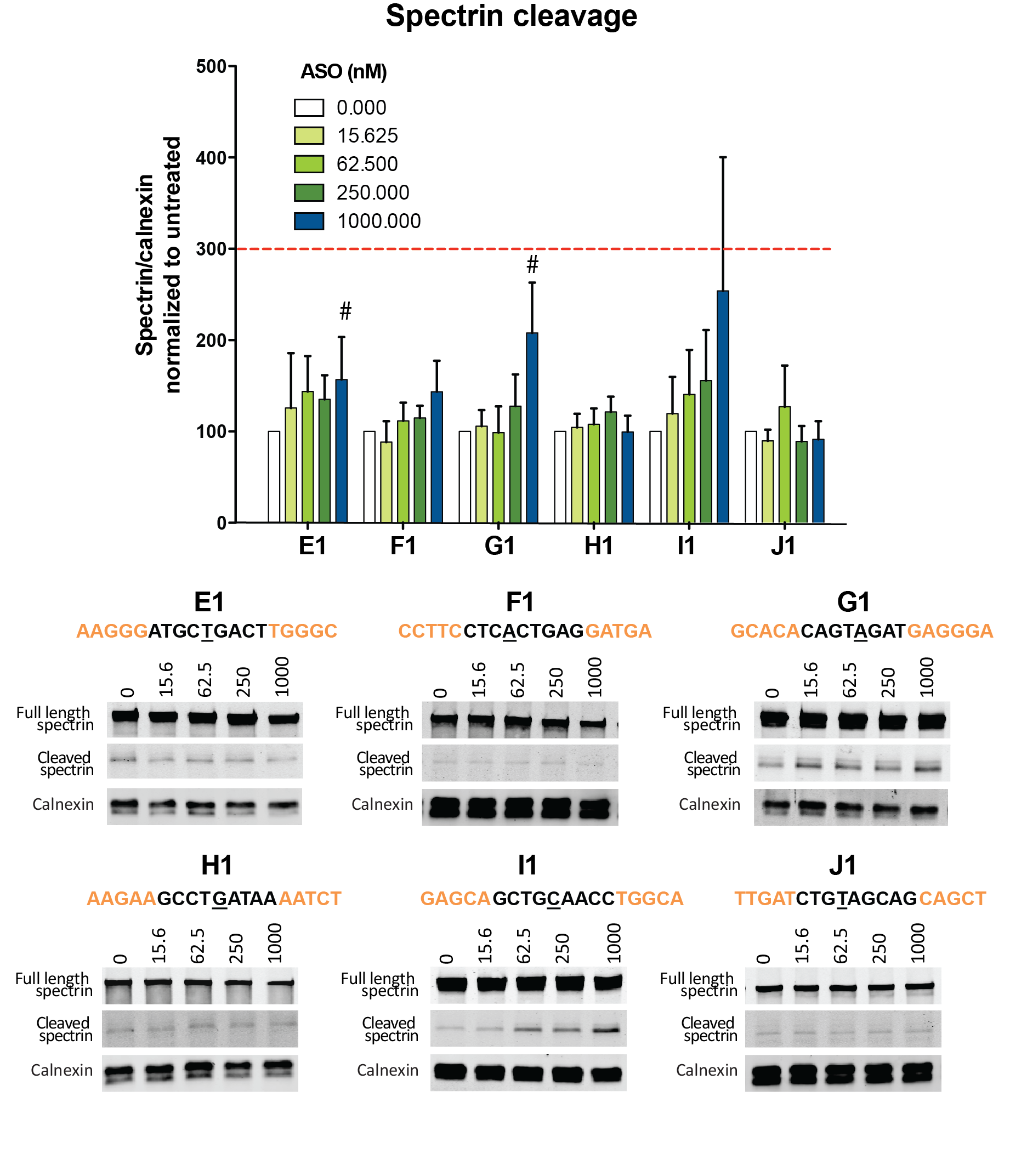

Supplement: Figure S3 — Selection of favourable SNP targets – Spectrin cleavage. Hu97/18 neurons were treated with 5e-9-5e ASOs targeted to 10 HD-SNPs and spectrin cleavage was analyzed. The 120 kDa fragment was normalized to calnexin and then to the untreated sample. HTT membranes were reprobed for spectrin. Representative images are shown. n = 4–6 per data point. Data are presented as mean ± SD. The # denotes two ASOs that induced rearrangement of the neurons. The PS backbone is represented by black; MOE modifications are illustrated by orange. The SNP is illustrated by the underlined nucleotide. The red dashed line represents the toxicity threshold. (TIF) [file pone.0107434.s003.tif]

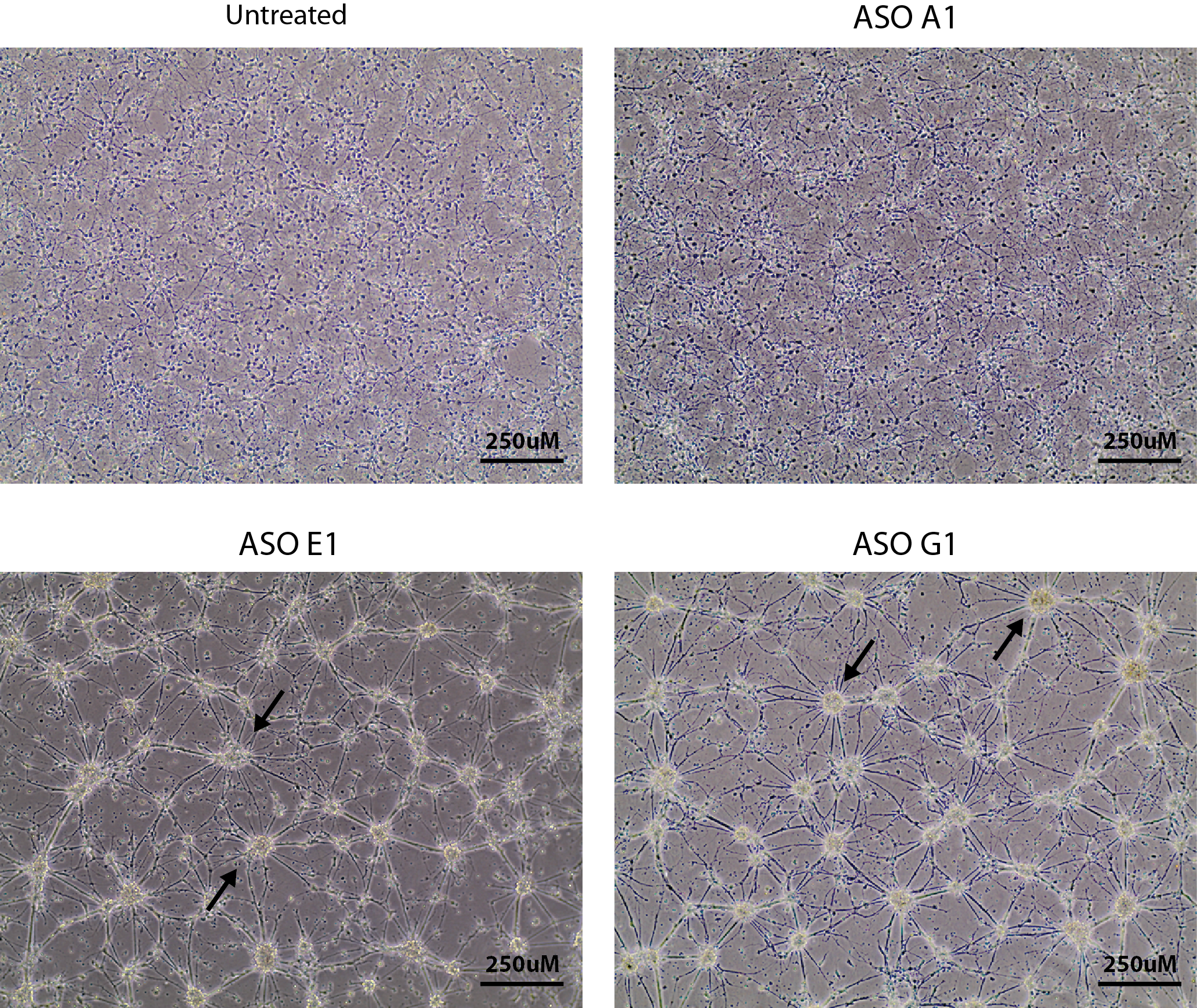

Supplement: Figure S4 — Altered neuronal morphology after treatment with some ASOs. Treatment with ASOs E1 and G1 caused marked morphological changes at the highest doses tested (1000 nM) resulting in rearrangement of neuronal cell bodies into an organized network. Representative images are shown of treated and untreated neurons. Black arrows indicate cell bodies grouped together connecting to other cell clusters. Images were taken with EVOS XL Core Imaging System from Life Technologies using the 10X objective. A calibration grid slide with 250 uM grids from MBF Bioscience was used to add a size marker to the images. (TIF) [file pone.0107434.s004.tif]

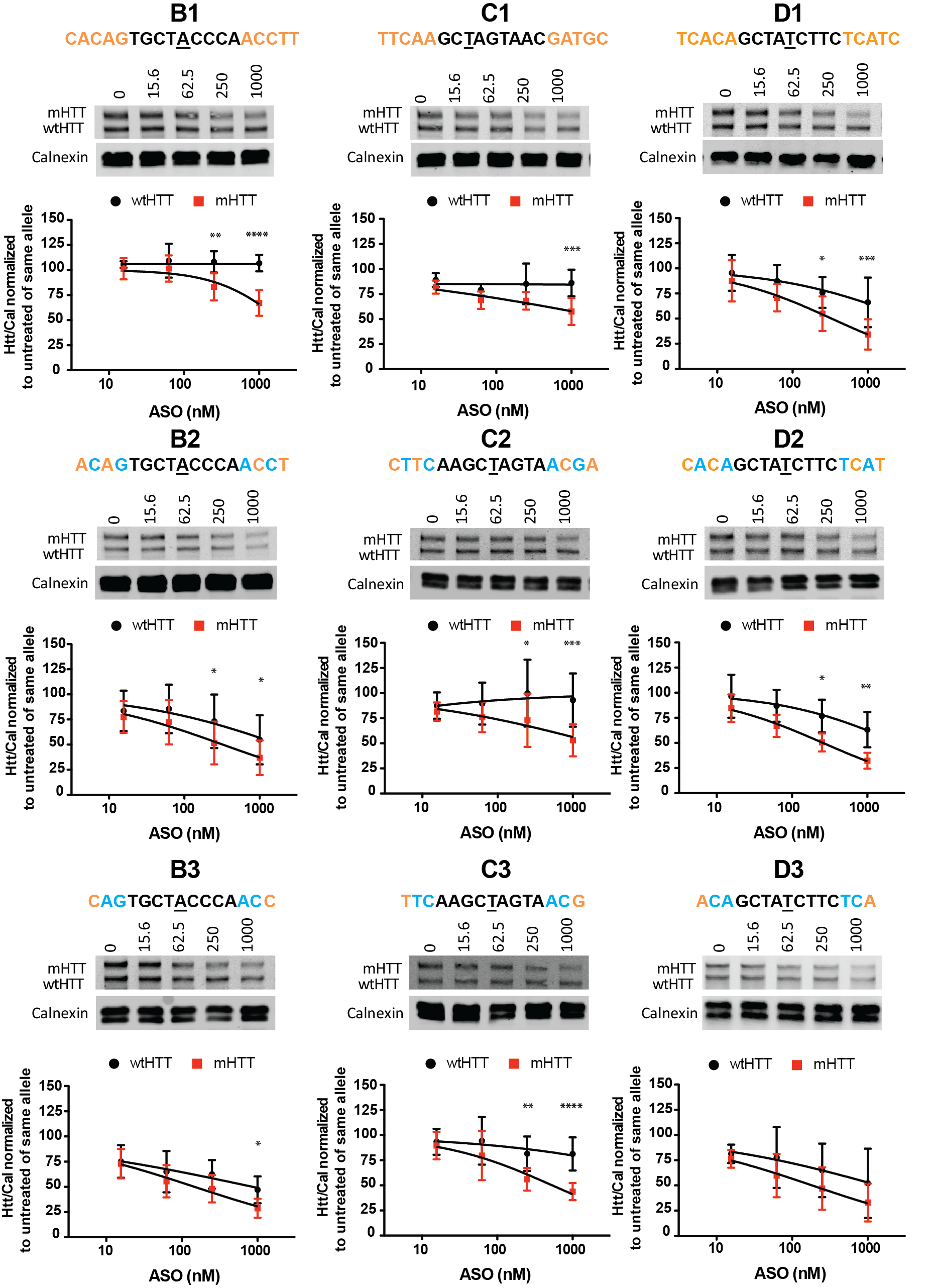

Supplement: Figure S5 — Targeting 4 SNPs using two different cEt motifs – HTT levels. Hu97/18 neurons were treated with ASOs with cEt modified wings and HTT protein was analyzed. HTT levels were normalized to calnexin and then to the untreated sample for each allele. Representative images are shown. n = 6–10 per data point. Data are presented as mean ± SD. The PS backbone is represented by black; MOE and cEt modifications are illustrated by orange and blue, respectively. The SNP is illustrated by the underlined nucleotide. (TIF) [file pone.0107434.s005.tif]

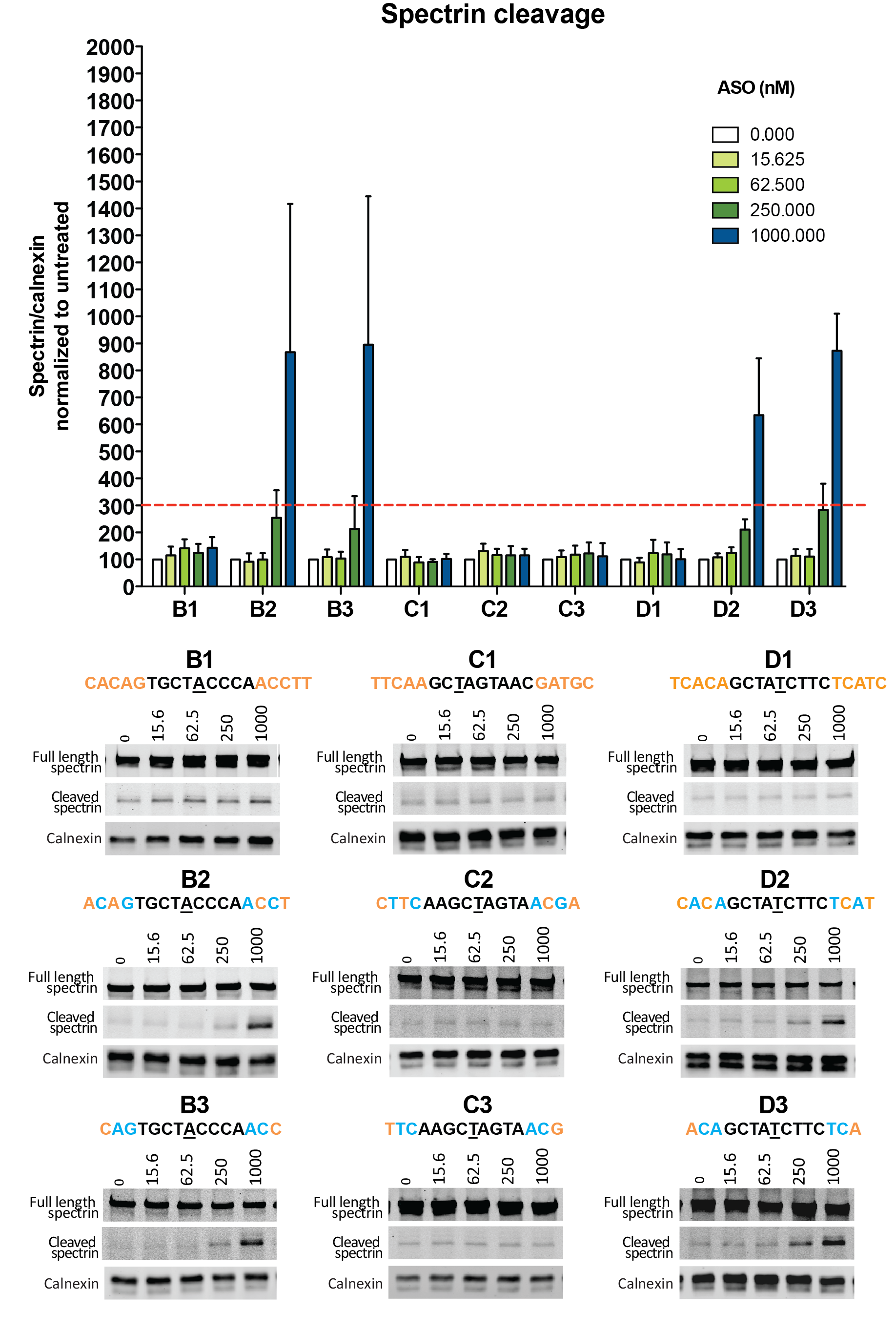

Supplement: Figure S6 — ASO screen at 4 SNPs using two different cEt motifs – Spectrin. Hu97/18 neurons were treated with ASO with cEt modified wings and spectrin cleavage was analyzed. The 120 kDa fragment was normalized to calnexin and then to the untreated sample. HTT membranes were reprobed for spectrin. Representative images are shown. n = 6–8 per data point. Data are presented as mean ± SD. The PS backbone is represented by black; MOE and cEt modifications are illustrated by orange and blue, respectively. The SNP is illustrated by the underlined nucleotide. The red dashed line represents the toxicity threshold. (TIF) [file pone.0107434.s006.tif]

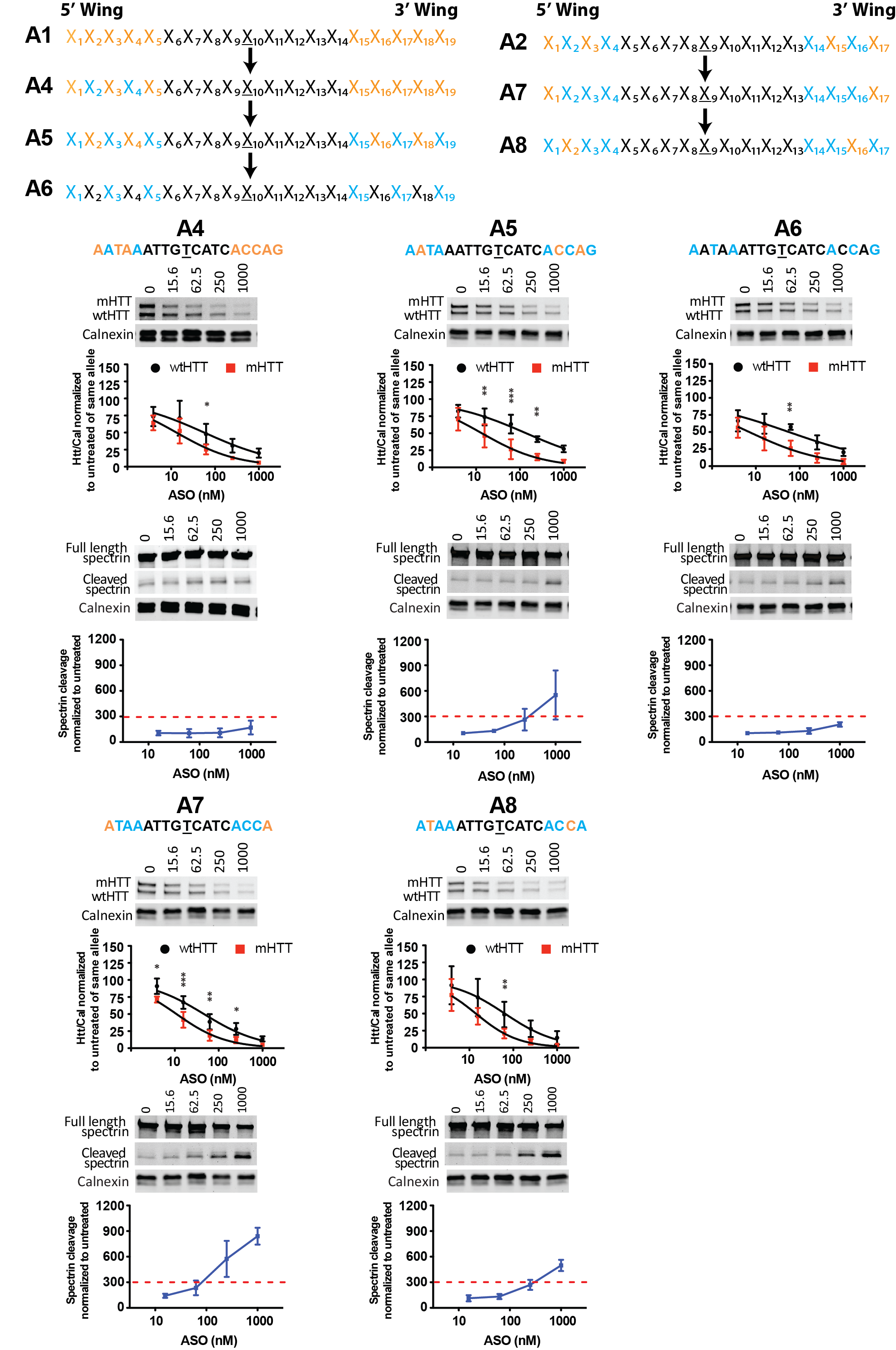

Supplement: Figure S7 — Wing SAR study. Hu97/18 neurons were treated with ASO with cEt modified wings and HTT protein and spectrin cleavage was analyzed. HTT levels were normalized to calnexin and then to the untreated sample for each allele. The 120 kDa fragment was normalized to calnexin and then to the untreated sample. Membranes were probed for HTT and reprobed for spectrin. Representative images are shown. n = 4–6 per data point. Data are presented as mean ± SD. The PS backbone is represented by black; MOE and cEt modifications are illustrated by orange and blue, respectively. The SNP is illustrated by the underlined nucleotide. The red dashed line represents the toxicity threshold. (TIF) [file pone.0107434.s007.tif]

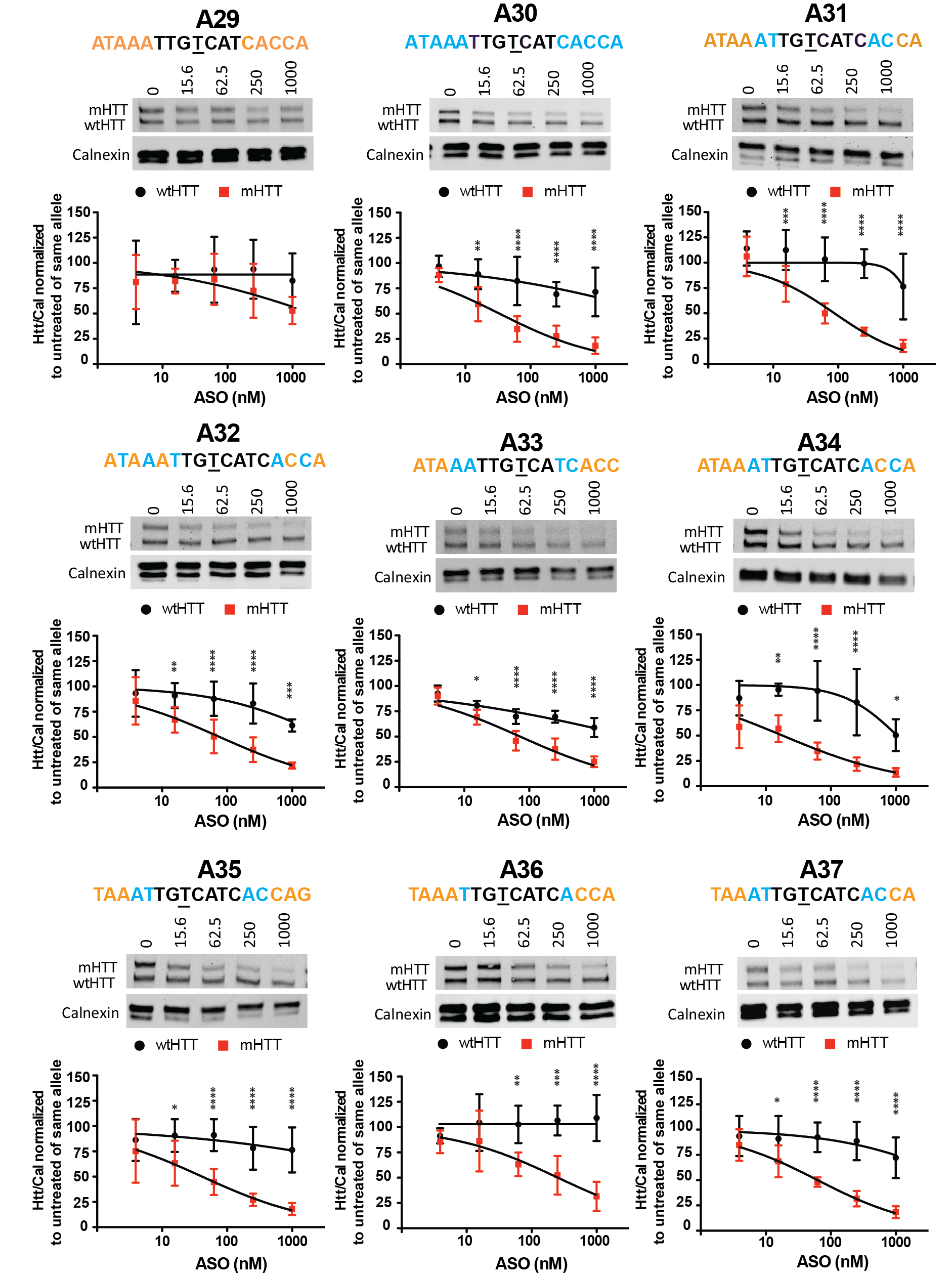

Supplement: Figure S8 — Shortening the gap to 7 nucleotides – HTT levels. Replacing PS-nucleotides with RNase H resistant nucleotides and shortening the gap increases selectivity by preventing cleavage at secondary cleavage sites and restricting cleavage to the main site next to the targeted SNP. Hu97/18 neurons were treated with ASOs and HTT protein was analyzed. HTT levels were normalized to calnexin and then to the untreated sample for each allele. Representative images are shown. n = 6–10 per data point. Data are presented as mean ± SD. The PS backbone is represented by black; MOE and cEt modifications are illustrated by orange and blue, respectively. The SNP is illustrated by the underlined nucleotide. (TIF) [file pone.0107434.s008.tif]

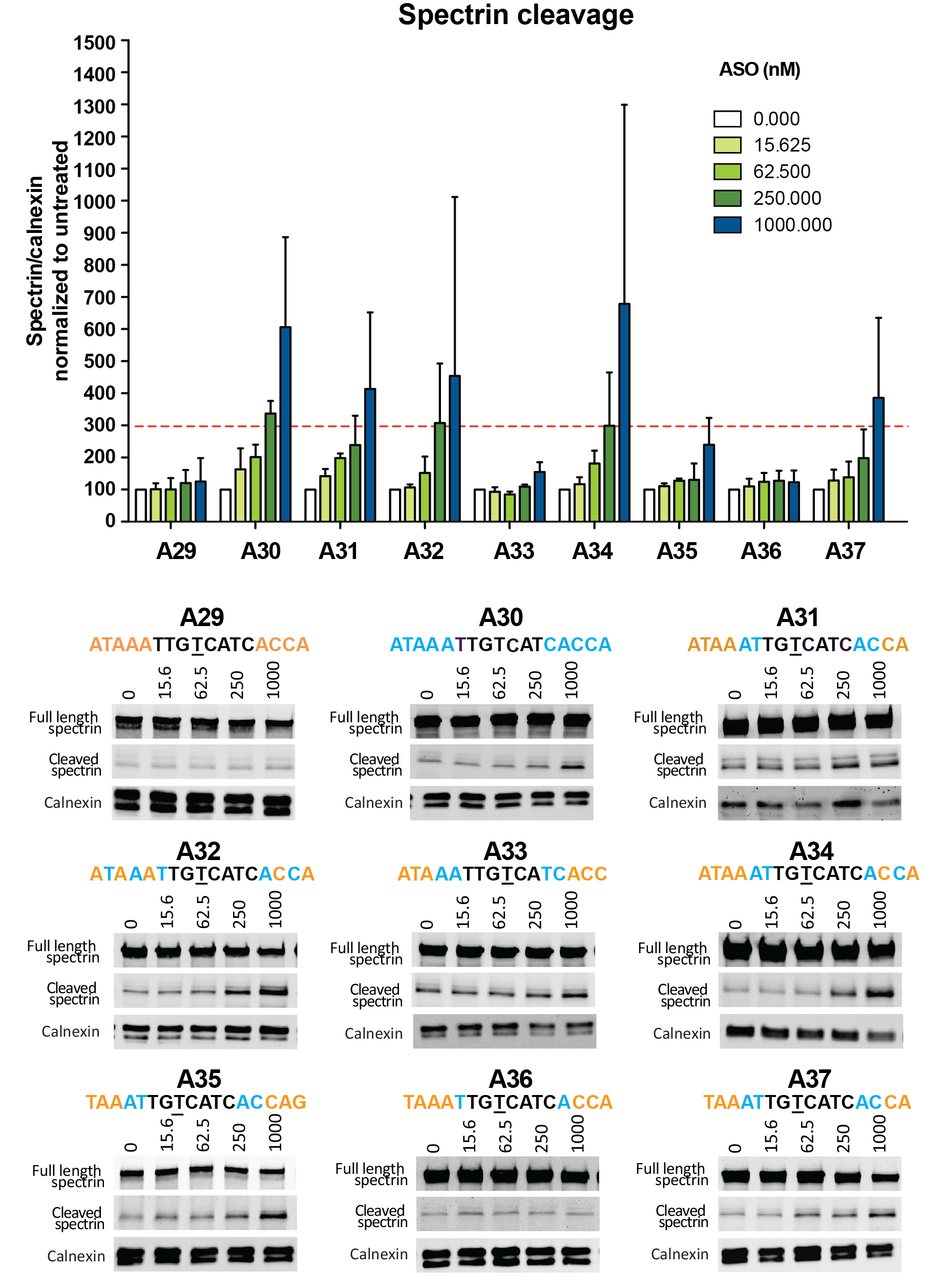

Supplement: Figure S9 — Shortening the gap to 7 nucleotides – Spectrin cleavage. Hu97/18 neurons were treated with ASOs and spectrin cleavage was analyzed. The 120 kDa fragment is normalized to calnexin and then to the untreated sample. HTT membranes were reprobed for spectrin. Representative images are shown. n = 6–8 per data point. Data are presented as mean ± SD. The PS backbone is represented by black; MOE and cEt modifications are illustrated by orange and blue, respectively. The SNP is illustrated by the underlined nucleotide. The red dashed line represents the toxicity threshold. (TIF) [file pone.0107434.s009.tif]
