# Supplementary material for: Allele-Specific Suppression of Mutant Huntingtin Using Antisense Oligonucleotides: Providing a Therapeutic Option for All Huntington Disease Patients
Source: PLoS One. 2014 Sep 10;9(9):e107434. doi: 10.1371/journal.pone.0107434 (PMC4160241; doi:10.1371/journal.pone.0107434)
Supplement: Table S1 — Summary of ASO RNA screen in human fibroblasts. MOE and cEt modifications are annotated by e and k, respectively. The SNP is underlined. (DOCX) [file pone.0107434.s010.docx]

**Supplemental table 1: Silencing of mHTT in human fibroblasts**

| **ASO** | **Notation** | **Rs #** | **Length** | **Gap-size** | **# cEt** | **#MOE** | **HTT levels** | | **Knock down ratio** |
| --- | --- | --- | --- | --- | --- | --- | --- | --- | --- |
|  |  |  |  |  |  |  | **% wtHTT** | **% mHTT** |  |
| **A3** | TeAkAkATTGTCATCAkCkCe | rs7685686 | 15 | 9 | 4 | 2 | 36 | 14 | 2.6 |
| **A9** | TeTkGkTkCATCACCAGAkAe | rs7685686 | 15 | 9 | 4 | 2 | 43 | 18 | 2.4 |
| **A10** | AeTkTkGkTCATCACCAGkAe | rs7685686 | 15 | 9 | 4 | 2 | 62 | 21 | 3.0 |
| **A11** | AeAkTkTkGTCATCACCAkGe | rs7685686 | 15 | 9 | 4 | 2 | 67 | 18 | 3.7 |
| **A12** | AeAkAkTkTGTCATCACCkAe | rs7685686 | 15 | 9 | 4 | 2 | 49 | 10 | 4.9 |
| **A13** | TeAkAkAkTTGTCATCACkCe | rs7685686 | 15 | 9 | 4 | 2 | 86 | 21 | 4.1 |
| **A14** | AeAkTkAkAATTGTCATCkAe | rs7685686 | 15 | 9 | 4 | 2 | 98 | 60 | 1.6 |
| **A15** | TeTkAkAkTAAATTGTCATkCe | rs7685686 | 15 | 9 | 4 | 2 | 86 | 78 | 1.1 |
| **A16** | AeTkTkAkATAAATTGTCkAe | rs7685686 | 15 | 9 | 4 | 2 | 137 | 129 | 1.1 |
| **A17** | TeAkTkTkAATAAATTGTkCe | rs7685686 | 15 | 9 | 4 | 2 | 105 | 93 | 1.1 |
| **A18** | GeTkCATCACCAGAkAkAkAe | rs7685686 | 15 | 9 | 4 | 2 | 55 | 28 | 2.0 |
| **A19** | TeGkTCATCACCAGkAkAkAe | rs7685686 | 15 | 9 | 4 | 2 | 62 | 18 | 3.4 |
| **A20** | TeTkGTCATCACCAkGkAkAe | rs7685686 | 15 | 9 | 4 | 2 | 45 | 13 | 3.5 |
| **A21** | AeTkTGTCATCACCkAkGkAe | rs7685686 | 15 | 9 | 4 | 2 | 66 | 22 | 3.0 |
| **A22** | AeAkTTGTCATCACkCkAkGe | rs7685686 | 15 | 9 | 4 | 2 | 68 | 17 | 4.0 |
| **A23** | AeAkATTGTCATCAkCkCkAe | rs7685686 | 15 | 9 | 4 | 2 | 35 | 8 | 4.4 |
| **A24** | TeAkAATTGTCATCkAkCkCe | rs7685686 | 15 | 9 | 4 | 2 | 45 | 12 | 3.8 |
| **A25** | AeTkAAATTGTCATkCkAkCe | rs7685686 | 15 | 9 | 4 | 2 | 91 | 62 | 1.5 |
| **A26** | AeAkTAAATTGTCAkTkCkAe | rs7685686 | 15 | 9 | 4 | 2 | 106 | 80 | 1.3 |
| **A27** | TeAkATAAATTGTCkAkTkCe | rs7685686 | 15 | 9 | 4 | 2 | 152 | 154 | 1.0 |
| **A28** | TeTkAATAAATTGTkCkAkTe | rs7685686 | 15 | 9 | 4 | 2 | 106 | 102 | 1.0 |

MOE (e) and cEt (k) modifications. The SNP is underlined.
